# *In silico* protein interaction screening uncovers DONSON’s role in replication initiation

**DOI:** 10.1126/science.adi3448

**Published:** 2023-09-22

**Authors:** Yang Lim, Lukas Tamayo-Orrego, Ernst Schmid, Zygimante Tarnauskaite, Olga V. Kochenova, Rhian Gruar, Sachiko Muramatsu, Luke Lynch, Aitana Verdu Schlie, Paula L. Carroll, Gheorghe Chistol, Martin A. M. Reijns, Masato T. Kanemaki, Andrew P. Jackson, Johannes C. Walter

**Affiliations:** 1 Department of Biological Chemistry and Molecular Pharmacology, Harvard Medical School, Blavatnik Institute; Boston, MA 02115, USA.; 2 MRC Human Genetics Unit, Institute of Genetics and Cancer, University of Edinburgh; Edinburgh, EH4 2XU, UK.; 3 Howard Hughes Medical Institute; Boston, MA 02115, USA.; 4 Department of Chromosome Science, National Institute of Genetics, Research Organization of Information and Systems (ROIS); Mishima, Shizuoka 411-8540, Japan.; 5 Biochemistry Department, Stanford School of Medicine; Stanford, CA 94305, USA.; 6 Chemical and Systems Biology Department, Stanford School of Medicine; Stanford, CA 94305, USA.; 7 Graduate Institute for Advanced Studies, SOKENDAI; Mishima, Shizuoka 411-8540, Japan.; 8 Department of Biological Science, The University of Tokyo; Tokyo 113-0033, Japan.

## Abstract

CDC45-MCM2-7-GINS (CMG) helicase assembly is the central event in eukaryotic replication initiation. In yeast, a multi-subunit “pre-Loading Complex” (pre-LC) accompanies GINS to chromatin-bound MCM2-7, leading to CMG formation. Here, we report that DONSON, a metazoan protein mutated in microcephalic primordial dwarfism, is required for CMG assembly in vertebrates. Using AlphaFold to screen for protein-protein interactions followed by experimental validation, we show that DONSON scaffolds a vertebrate pre-LC containing GINS, TOPBP1, and DNA pol ε. Our evidence suggests that DONSON docks the pre-LC onto MCM2-7, delivering GINS to its binding site in CMG. A patient-derived DONSON mutation compromises CMG assembly and recapitulates microcephalic dwarfism in mice. These results unify our understanding of eukaryotic replication initiation, implicate defective CMG assembly in microcephalic dwarfism, and illustrate how *in silico* protein-protein interaction screening accelerates mechanistic discovery.

Rapid and faithful DNA replication is necessary for cell proliferation and the maintenance of genome integrity, and its disruption causes cancer and many inherited human diseases. A key component of the replisome is the replicative CMG helicase, which is composed of CDC45, MCM2-7, and GINS, and unwinds DNA at the replication fork. CMG assembly involves several discrete steps and is best understood in yeast (Fig. 1A) (1). In the G1 phase, the hexameric MCM2-7 ATPase is loaded onto origins of replication in a head-to-head orientation (“double hexamers”), a process called licensing. Subsequently, the Sld3-Sld7 complex binds to Dbf4-dependent kinase (DDK)-phosphorylated MCM2-7 double hexamers on chromatin and recruits Cdc45. In parallel, CDK phosphorylation of Sld2 mediates Sld2 binding to Dpb11, promoting the assembly of a “pre-loading complex” (pre-LC) that also contains GINS and Pol ε (2–5). CDK also phosphorylates Sld3, and binding of Dpb11 to phosphorylated Sld3 allows docking of the pre-LC onto Cdc45-MCM2-7, delivering GINS for stable CMG assembly. Notably, Cdc45 binds MCM2-7 only weakly until GINS is recruited, when a stable CMG complex forms (5–7). Once assembled, CMGs are activated for DNA unwinding by MCM10, followed by replisome assembly and bi-directional DNA replication. Despite these insights, a molecular understanding of how the pre-LC promotes GINS association with Cdc45-MCM2-7 is still lacking, even in yeast, primarily because of the absence of relevant structural information (1).

The mechanism of CMG assembly in metazoans is broadly similar to that observed in yeast. TRESLIN and MTBP are thought to perform the same role in Cdc45 recruitment as their yeast counterparts, Sld3 and Sld7 (Fig. 1B) (8–12). Furthermore, analogous to Sld3’s interaction with Dpb11, CDK-phosphorylated TRESLIN binds to Dpb11’s ortholog, TOPBP1 (8, 13–15). TOPBP1 in turn contacts GINS (16). However, a central mystery concerns the metazoan counterpart of Sld2 because RECQL4, the closest vertebrate homolog of Sld2, functions after CMG assembly (17, 18). Furthermore, although Pol ε is an essential component of the pre-LC in yeast, this polymerase is dispensable for vertebrate CMG assembly (19, 20). Finally, there remain several unanswered questions including whether a vertebrate pre-LC exists, how it might be organized, and how GINS is delivered in vertebrates.

Microcephalic dwarfism comprises a family of monogenic disorders of extreme growth failure that result from disruption of cellular proliferation (21). Many genes implicated in microcephalic dwarfism act in DNA replication and encode licensing factors, components of the CMG helicase, DNA polymerases, and replication stress response factors (22). Mutations in the Downstream Neighbor of SON (DONSON) protein also cause microcephalic dwarfism, including Meier-Gorlin syndrome, a disorder specifically associated with replication initiation genes (23, 24). Phenotypic analysis in *Drosophila* suggested that DONSON plays a role in DNA replication (25). Similar to the fly protein, human DONSON expression peaks in S phase. It also localizes to sites of replication and co-immunoprecipitates with several replisome components, including CMG components (23, 26). However, in mammals, studies using small interfering RNA (siRNA) and patient-derived cell lines suggested roles for DONSON in maintaining replication fork stability, ATR signaling, and replicative traverse of DNA interstrand crosslinks (23, 26). Thus, a clear picture of DONSON’s role in genome maintenance has not emerged. Here, we show that DONSON organizes a vertebrate pre-LC that delivers GINS to its binding site in CMG and we implicate defective CMG assembly in the pathophysiology of microcephalic dwarfism.

## Results

### DONSON is required for CMG assembly in frog egg extracts

**Fig. 1.**
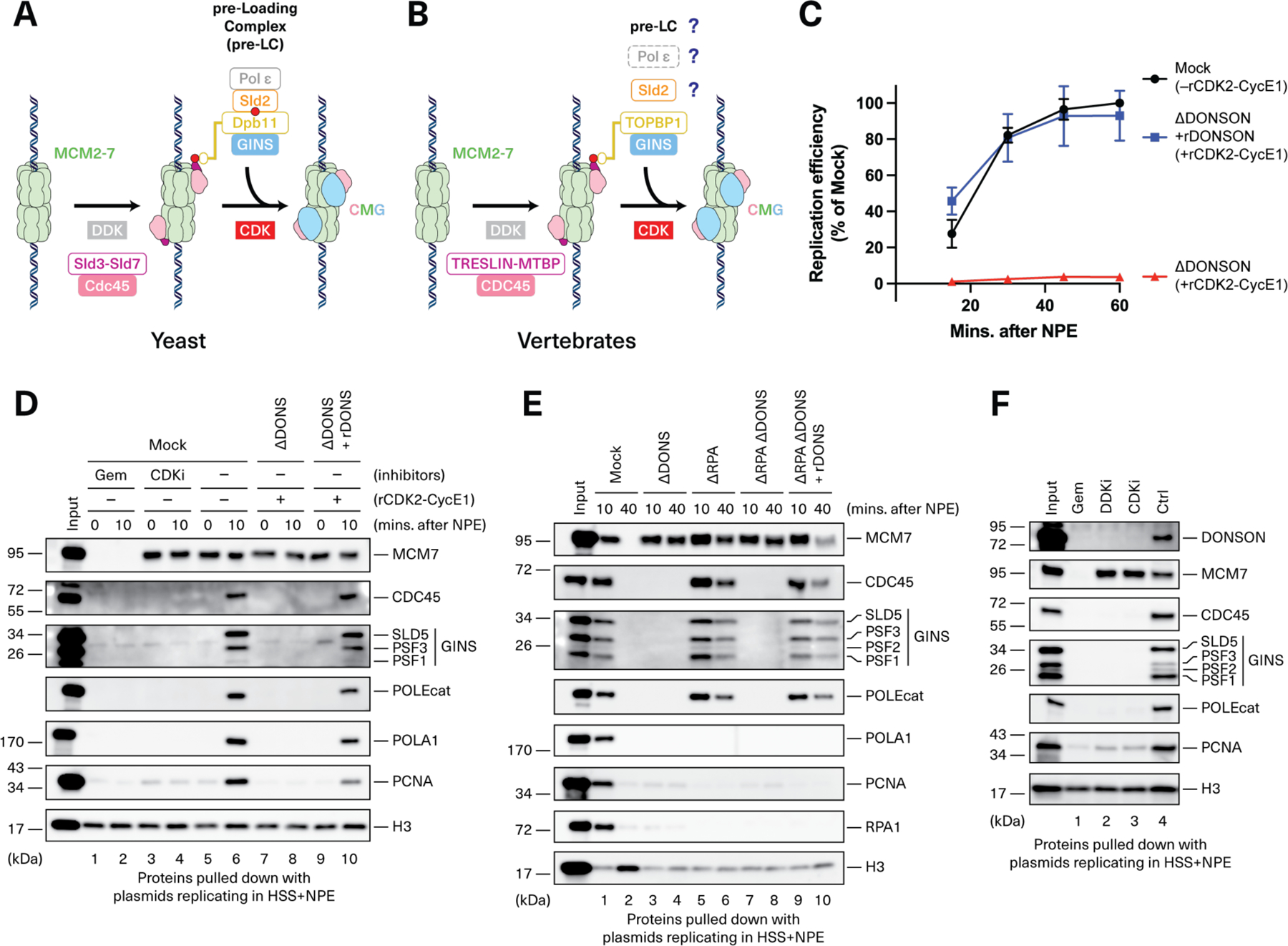
DONSON is required for CMG assembly and DNA replication. **(A-B)** Models of CMG assembly in budding yeast and vertebrates. **(C)** Relative DNA replication efficiency in the indicated egg extracts. Because depletion of DONSON co-depletes roughly half of the 0.5 to 1 µM endogenous CDK2-Cyclin E, DONSON-depleted (ΔDONSON) extracts but not mock-depleted extracts were supplemented with 0.3 µM recombinant human CDK2-Cyclin E1. Recombinant DONSON (rDONSON, fig. S1B) was added where indicated. Datapoints, n=3 experiments. Mean±SD. A representative western blot of total protein levels in these reactions is shown in fig. S1E. **(D)** Plasmid DNA was incubated in the indicated egg extracts. At the specified times following NPE addition, chromatin was recovered and blotted for the indicated proteins. DONS, DONSON; Gem, geminin; CDKi, p27^Kip^. A representative western blot of total protein levels in these reactions is shown in fig. S1E. **(E)** Plasmid DNA was incubated in extracts depleted of DONSON and/or RPA. At the specified times following NPE addition, chromatin was recovered and blotted for the indicated proteins. Western blot of total protein levels in these reactions is shown in fig. S1H. **(F)** In the presence of the indicated inhibitors of replication initiation, plasmid DNA was recovered 10 minutes after NPE addition and blotted for the indicated proteins. DDKi, PHA-767491.

To assess DONSON’s role in genome maintenance, we used nucleus-free *Xenopus laevis* egg extracts, which faithfully recapitulate DNA replication and the replication stress response (27). Plasmid DNA is first incubated with a high-speed supernatant (HSS) of total egg lysate, which promotes replication licensing (fig. S1A). Subsequent addition of a concentrated nucleoplasmic extract (NPE) leads to CMG assembly and a complete round of DNA replication that can be monitored through [α−^32^P]dATP incorporation. Immunodepletion of DONSON from HSS and NPE abolished DNA replication, which was partially rescued by re-addition of bacterially-expressed DONSON (fig. S1B and C). Partial rescue was explained by the fact that depletion of DONSON co-depleted roughly half of the CDK2-Cyclin E (fig. S1D to E), which is rate-limiting for replication in nucleus-free egg extracts (27–30). Indeed, when we supplemented DONSON-depleted extracts with recombinant CDK2-Cyclin E1 (rCDK2-Cyclin E1) (fig. S1E), recombinant DONSON fully rescued replication (Fig. 1C). Thus, DONSON is required for vertebrate, cell-free DNA replication.

To determine which replication step is dependent on DONSON, we performed chromatin pull-down experiments. As shown in Fig. 1D, licensing, as measured by MCM7 chromatin binding, was unaffected by DONSON depletion or add-back (lanes 5, 7, and 9). Moreover, TRESLIN-MTBP recruitment did not depend on DONSON, suggesting that DONSON is not required for initial steps in CMG assembly (fig. S1F, lanes 6 and 8; fig. S1G, lanes 8 and 10). By contrast, CDC45, GINS, Pol ε, Pol α, and proliferating cell nuclear antigen (PCNA) recruitment– which occurred within 10 minutes of NPE addition–failed in DONSON-depleted extract, but their recruitment was restored with recombinant DONSON (Fig. 1D, lanes 6, 8, and 10). DONSON depletion also abolished CMG assembly in extracts lacking replication protein A (RPA) (Fig. 1E, lanes 7 and 8). RPA is required for origin unwinding and replication elongation (31, 32), as seen from defective PCNA and Pol α loading and the persistence of CDC45, GINS, and Pol ε on chromatin at the 40-min point (Fig. 1E, lanes 5 and 6; histone H3 loading is low compared with mock-depleted extract due to deficient replication). Thus, DONSON is required for *de novo* CMG assembly, independently of any effects on CMG stability during the subsequent, RPA-dependent unwinding and elongation phases of replication initiation. The defects seen upon DONSON depletion mirrored the effect of adding the CDK2 inhibitor p27^Kip^ (33, 34) (Fig. 1D, lane 4, CDKi), suggesting that DONSON functions at the final stage of CMG assembly. Consistent with this model, DONSON binding to chromatin was blocked by CDKi, DDKi, and geminin, an inhibitor of origin licensing (Fig. 1F). Collectively, our results show that frog DONSON functions after TRESLIN-MTBP loading onto licensed chromatin but before assembly of a stable CMG helicase containing GINS and CDC45.

### A model for DONSON function based on *in silico* protein-protein interaction screening

**Fig. 2.**
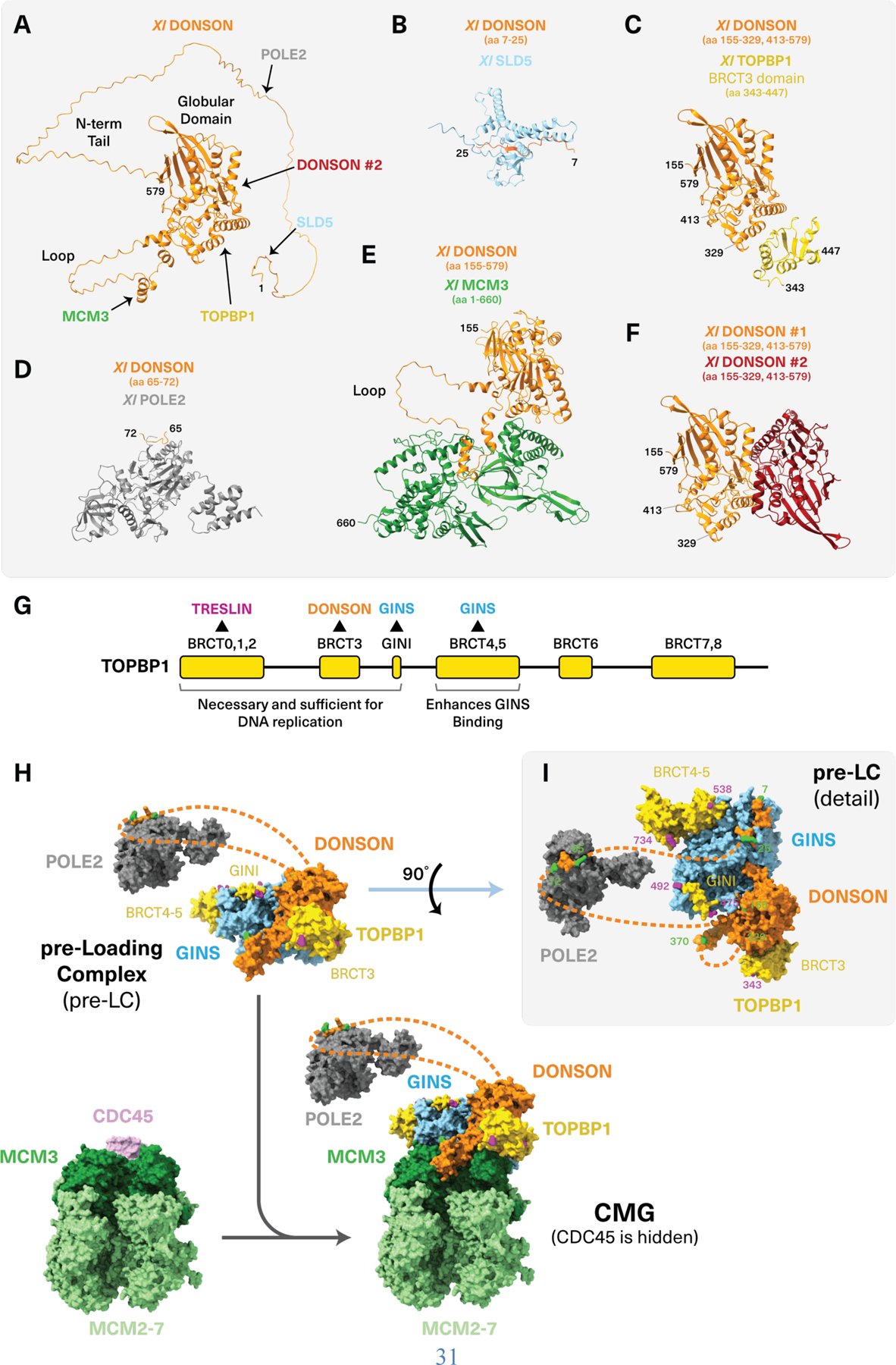
Hypothetical model of DONSON function in CMG assembly. **(A)** AlphaFold-Multimer (AF-M) prediction of DONSON’s structure. Sites predicted to bind interacting proteins are indicated with arrows. All proteins shown are from *Xenopus*, but the predicted human complexes appear almost identical. **(B** to **F)** AF-M predictions of relevant DONSON domains complexed with SLD5 (B), TOPBP1 (C), POLE2 (D), MCM3 (E), and a second copy of DONSON (F). The amino acids (aa) of each protein shown are indicated in brackets. **(G)** Functional domains of TOPBP1. **(H)** AF-M predictions suggest that a pre-LC consisting of DONSON, GINS, Pol ε, and TOPBP1 docks onto the MCM2-7 complex through the predicted DONSON-MCM3 interaction. **(Top)** The predicted *Xenopus* pre-LC is shown with only the POLE2 subunit of Pol ε and just the BRCT3 (aa 343–447), GINI (aa 475–492), and BRCT4-5 (aa 538–734) domains of TOPBP1. Disordered regions of DONSON are shown as dotted lines but omitted for TOPBP1. Residues located at the ends of well-ordered segments are shown in green and purple for DONSON and TOPBP1, respectively. **(Bottom)** The pre-LC was docked onto the cryo-EM structure of human CMG [PDB: 7PLO (66)] by aligning on MCM3. Only CDC45 and MCM2-7 of human CMG are shown. See Methods for modeling details. **(I)** The pre-LC from (H) rotated by 90 degrees. Residues located at the ends of well-ordered segments are numbered and shown in green and purple for DONSON and TOPBP1, respectively.

We next used recent advances in structure prediction to address how DONSON promotes CMG assembly. AlphaFold2 predicts that DONSON contains a ~150-residue disordered N-terminal tail, a globular domain, and an ~80-residue loop protruding from the globular domain (Fig. 2A). However, this structure on its own offers no insight into DONSON function. We therefore used AlphaFold-Multimer (AF-M) (35) to screen *in silico* for potential DONSON interactors among a common set of ~70 core DNA replication factors in humans, frogs, worms, and flies. Based on confidence metrics generated by AlphaFold, the top proteins predicted to interact with DONSON in all four species included SLD5 (a GINS subunit), TOPBP1, POLE2 (a Pol ε subunit), and MCM3 (a MCM2-7 subunit), all of which are implicated in CMG assembly [Fig. 2, B to E; table S1 for AlphaFold confidence values; fig. S2 for structures colored by local distance difference test (pLDDT) values; fig. S3 for predicted alignment error plots]. DONSON was also strongly predicted to interact with itself (Fig. 2F and fig. S3F). Thus, AF-M–based *in silico* screening was consistent with DONSON functioning during CMG assembly.

AF-M predicted that DONSON binds TOPBP1, MCM3, POLE2, SLD5, and itself through five distinct regions as follows: DONSON’s disordered N-terminus was predicted to bind SLD5 (Fig. 2B and fig. S3A) and when DONSON was folded with the tetrameric GINS complex, the interaction was extended to DONSON’s globular domain (Fig. 2I and fig. S3B) and the confidence of the interaction increased in most organisms (table S1). An adjacent disordered DONSON peptide was predicted to bind POLE2 (Fig. 2D and fig. S3D). Binding to the AAA+ domain of MCM3 was predicted to involve the flexible loop that protrudes from DONSON’s globular domain (Fig. 2E and fig. S3E). DONSON’s globular domain was predicted to bind the BRCT3 domain of TOPBP1 that is essential for DNA replication (Fig. 2, C and G, and fig. S3C) (8). Finally, another part of the globular domain was predicted to mediate dimerization (Fig. 2F and fig. S3F). Notably, AF-M predicted that DONSON can contact all its potential binding partners simultaneously (figs. S4 and S5 and data S1). When folded with two copies of DONSON, the TOPBP1 BRCT3 domain was predicted to bind at the DONSON dimer interface (fig. S4, A and D, and data S1), suggesting that it might stabilize a DONSON dimer.

Based on these *in silico* results, we hypothesized that DONSON organizes a vertebrate pre-LC that includes GINS, TOPBP1, and Pol ε (Fig. 2H, top, and Fig. 2I). We further postulated that this pre-LC docks onto MCM3 to deliver GINS to the CDC45-MCM2-7 complex (Fig. 2H). We confirmed DONSON dimerization using mass photometry (fig. S6). This observation suggests that the pre-LC dimerizes (see Discussion) but for simplicity it is depicted as a monomer. When the pre-LC was docked onto the cryo-electron microscopy (cryo-EM) structure of the replisome through the predicted DONSON-MCM3 interaction, GINS from the pre-LC aligned well with GINS on CMG [root mean square deviation (RMSD) = 5.3 Å, fig. S7]. Thus, our modeling suggests that DONSON promotes CMG assembly by delivering GINS directly to its binding site on CDC45-MCM2-7 (Fig. 2H).

### DONSON organizes a pre-LC containing GINS, TOPBP1, and Pol ε

**Fig. 3.**
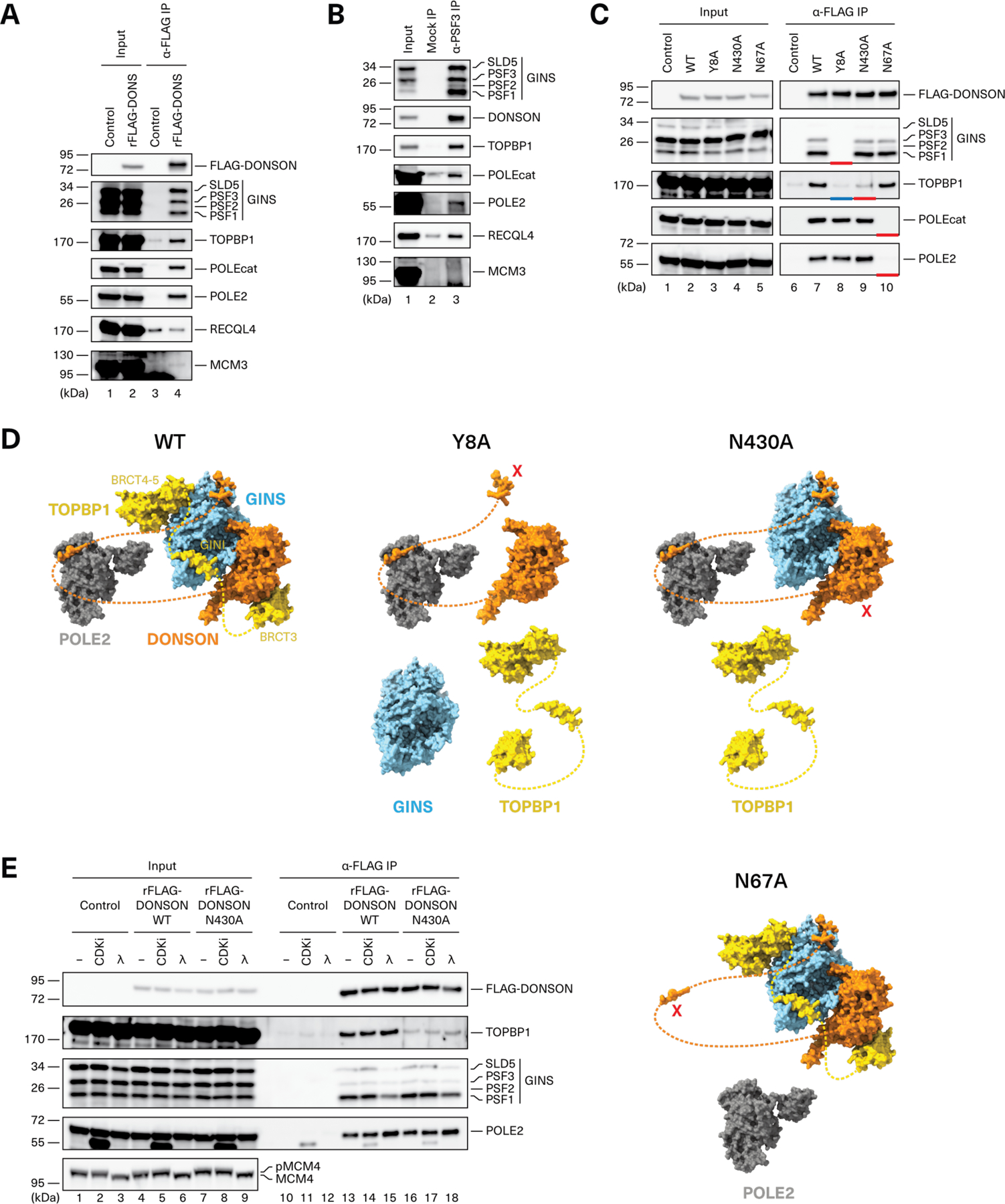
DONSON forms a pre-LC. **(A)** Recombinant FLAG-tagged DONSON (rFLAG-DONSON) was added to non-replicating nucleoplasmic egg extract (NPE), recovered, and blotted for the indicated proteins alongside the input extract. **(B)** Endogenous GINS was immunoprecipitated from NPE using PSF3 antibody and blotted for the indicated proteins. **(C)** rFLAG-DONSON proteins containing specified mutations (figs. S1B and S11A) were added to NPE, recovered, and blotted as indicated. Red and blue bars show missing pre-LC components. The images are part of the same western blot, which was cropped to remove irrelevant information between lanes 5 and 6. **(D)** The effects of different DONSON mutants are depicted in the context of the AF-M-modeled pre-LC (as in Fig. 2I). Mutations are indicated as red Xs. **(E)** The indicated rFLAG-DONSON proteins were added to NPE treated with buffer, p27^Kip^ (CDKi), or λ phosphatase. DONSON was recovered and blotted for the indicated proteins. Total extract was also blotted for MCM4 to show λ phosphatase activity.

To test the model presented in Fig. 2, we investigated which factors interact with DONSON in nonreplicating nucleoplasmic extract. Recombinant FLAG-tagged DONSON (fig. S1B, right panel) was added to extract and immunoprecipitated (IP’ed). FLAG-DONSON co-IP’ed GINS, TOPBP1, POLE2, and POLEcat but not RECQL4 or MCM3 (Fig. 3A). Reciprocal IP of endogenous GINS recovered DONSON, TOPBP1, POLE2 and POLEcat (Fig. 3B). We conclude that, independently of DNA replication, DONSON forms a stable pre-LC with GINS, TOPBP1, and Pol ε, but not with MCM3 or RECQL4.

We next probed the architecture of the pre-LC using site-directed mutagenesis. Residues Y8, N430, and N67 in DONSON were predicted to interact with GINS, TOPBP1, and POLE2, respectively (fig. S8, A to C). Indeed, mutation of each residue to alanine led to defective co-IP of GINS, TOPBP1, and Pol ε, respectively (Fig. 3C, lanes 8 to 10, red bars; Fig. 3D). DONSON^Y8A^ failed to co-IP not only GINS, as predicted, but also TOPBP1 (Fig. 3C, blue bar, and Fig. 3D). This suggests that TOPBP1 binds cooperatively to DONSON and GINS. Consistent with this idea, TOPBP1 binds GINS through two elements, a short “GINI” peptide located in a disordered region of TOPBP1 and the BRCT4-5 domains located C terminal to the GINI peptide (Fig. 2G and fig. S9, A to C) (16, 36). Unlike the GINI peptide, BRCT4-5 is not essential for DNA replication but it stabilizes the interaction of TOPBP1 with GINS (16, 36) and DONSON (fig. S10). BRCT4-5 binds the same site on GINS that is occupied by POLE2 in the fully assembled replisome (fig. S9D)(36). We therefore propose that within the pre-LC, TOPBP1’s BRCT4-5 and GINI domains occupy the POLE2 binding site on GINS. Furthermore, our observation that DONSON interacts with Pol ε independently of GINS and TOPBP1 (Fig. 3C, lane 8) suggests that Pol ε is flexibly tethered by POLE2 to the pre-LC through DONSON’s disordered N-terminal tail (Fig. 2H) and that it associates with GINS only after CMG assembly.

We next asked whether purified DONSON, GINS, and TOPBP1 are sufficient to form a complex. We omitted Pol ε because its association with the pre-LC is not essential for CMG assembly [(19, 20) see below]. Indeed, purified DONSON^WT^ co-IP’ed TOPBP1^1−530^ and GINS, whereas DONSON^Y8A^ did not, and DONSON^N430A^ IP’ed GINS but recovered little TOPBP1 (fig. S11B, lanes 22 to 24), as seen in extracts (Fig. 3C). Furthermore, DONSON^WT^ and DONSON^N430A^–but not DONSON^Y8A^–bound efficiently to GINS in the absence of TOPBP1 (fig. S11B, lanes 14 to 16). We conclude that DONSON, GINS, and TOPBP1 are sufficient to form the core of a vertebrate pre-LC that also associates with Pol ε. Pre-LC formation appears to be independent of CDK activity (Fig. 3E), consistent with the fact that the essential TOPBP1 BRCT domain that contacts DONSON (BRCT3) is not predicted to bind phospho-peptides (37). These biochemical experiments provide powerful support for the pre-LC architecture predicted by AF-M.

### DONSON binding to GINS and TOPBP1, but not Pol ε, is required for CMG assembly

**Fig. 4.**
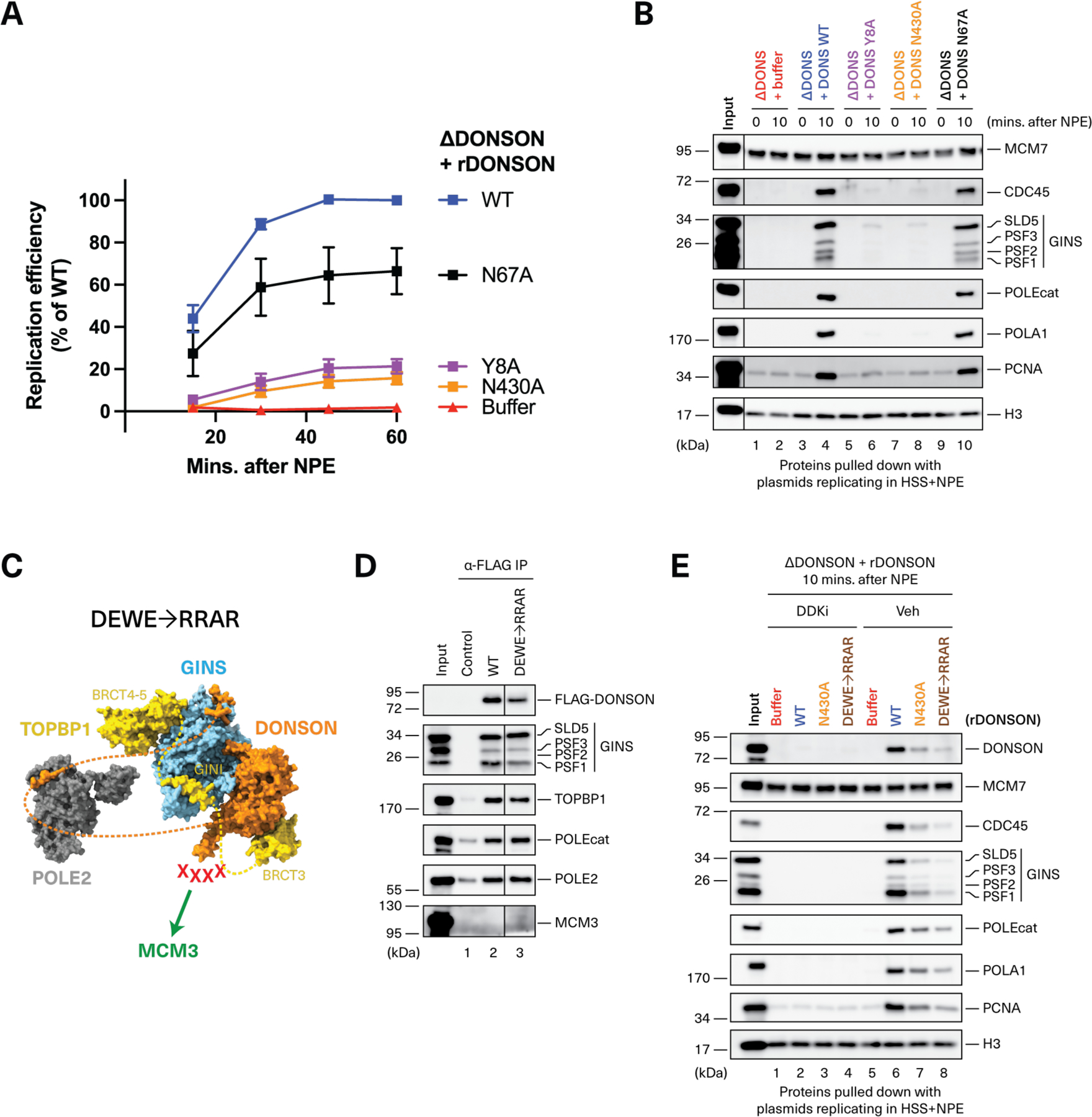
Pre-LC formation is required for DNA replication. **(A)** Egg extracts were depleted of DONSON, supplemented with rCDK2-Cyclin E1 and the indicated DONSON proteins (figs. S1B and S11A), and used to measure DNA replication. Datapoints, *n* = 4 experiments, except N67A where *n* = 3. Mean ± SD. **(B)** Plasmid pull-down (as in Fig. 1D) to assay the effect of DONSON mutations on CMG assembly. The images are part of the same western blot, which was cropped to remove irrelevant information between the input and lane 1. Western blot of total protein levels in these reactions is shown in fig. S11C. **(C)** DONSON^DEWE→RRAR^, depicted as in Fig. 3D. **(D)** FLAG-DONSON immunoprecipitation (as in Fig. 3A) showing that DONSON^DEWE→RRAR^ (expressed in wheat germ extract) is proficient in pre-LC assembly. The images are part of the same western blot, which was cropped to remove irrelevant information between lanes 2 and 3. **(E)** Plasmid pull-down (as in Fig. 1F) showing that purified recombinant DONSON^N430A^ and DONSON^DEWE→RRAR^ (fig. S11A) bind inefficiently to chromatin during replication. Western blot of total protein levels in these reactions is shown in fig. S11D.

We next assessed whether pre-LC assembly is required for DNA replication. DONSON^Y8A^ (defective in GINS and TOPBP1 recruitment) did not support efficient DNA replication (Fig. 4A) or CMG assembly (Fig. 4B, lane 6). Additional DONSON mutations at the predicted DONSON-GINS interface provided further evidence that poor GINS binding correlates with inefficient DNA replication (fig. S12). To perturb the other side of the DONSON-GINS interface, we mutated histidine 76 in the SLD5 subunit of GINS, which is predicted to contact Y8 in DONSON (fig. S13, A and B). As shown in fig. S13, C and D, GINS^SLD5-H76A^ co-IP’ed DONSON weakly and supported only low levels of DNA replication. Thus, DONSON’s interaction with GINS is essential for CMG assembly and efficient DNA replication.

We performed a similar analysis for the DONSON-TOPBP1 interaction. DONSON^N430A^ (defective in TOPBP1 recruitment) supported inefficient CMG assembly and DNA replication (Fig. 4, A and B). Additional DONSON mutations predicted to disrupt the DONSON-TOPBP1 interaction also compromised TOPBP1 co-IP and DNA replication (fig. S14). Conversely, TOPBP1 mutations engineered at the predicted DONSON-TOPBP1 interface showed a correlation between poor DONSON binding and inefficient DNA replication (fig. S15). These results indicate that, similar to the DONSON-GINS interaction, the DONSON-TOPBP1 interaction is required for CMG assembly and DNA replication.

By contrast, DONSON^N67A^, which failed to bind Pol ε (Fig. 3C), supported almost normal levels of CMG assembly and DNA replication (Fig. 4, A and B), consistent with previous studies showing CMG formation in Pol ε-deficient egg extracts and human cells (19, 20). This result is also consistent with Pol ε being loosely associated with the pre-LC through DONSON, and binding tightly to the replisome only after CMG has been assembled. Together, our data indicate that DONSON, GINS, and TOPBP1 form the core of an essential, vertebrate pre-LC that chaperones GINS onto chromatin-bound MCM2-7. Unlike yeast, the vertebrate pre-LC contains DONSON instead of Sld2; Pol ε, though present, is dispensable for CMG assembly.

### DONSON’s predicted MCM3 binding domain is necessary for CMG assembly

AF-M predicts with high confidence that an α-helix on DONSON’s flexible loop interacts with MCM3 (Fig. 2E, fig. S3E, and table S1). To test whether this predicted interaction delivers GINS to the chromatin (Fig. 2H), we mutated three acidic residues (D374, E377, E384) and a highly conserved tryptophan (W381) to arginines and alanine, respectively (fig. S8D). All four mutations were located in the MCM3-binding helix and together generated DONSON^DEWE→RRAR^ (Fig. 4C). Although DONSON^DEWE→RRAR^ was fully competent for pre-LC assembly (Fig. 4D), it was deficient in DDK-dependent chromatin-binding and CMG assembly (Fig. 4E, lane 8), and supported inefficient DNA replication (fig. S11E). Because MCM3 did not co-IP with DONSON from nonreplicating egg extract (Fig. 3A and Fig. 4D), we infer that stable binding of DONSON to MCM3 only occurs after TOPBP1 tethers the pre-LC to MCM double hexamers (see Discussion). Consistent with this idea, DONSON^N430A^, which did not bind TOPBP1 efficiently (Fig. 3C), was similarly defective as DONSON^DEWE→RRAR^ in chromatin binding (Fig. 4E, lane 7). Thus, our evidence is consistent with the predicted DONSON-MCM3 interaction being important to deliver the pre-LC to chromatin for CMG assembly.

### DONSON is required for CMG assembly in mammalian cells

**Fig. 5.**
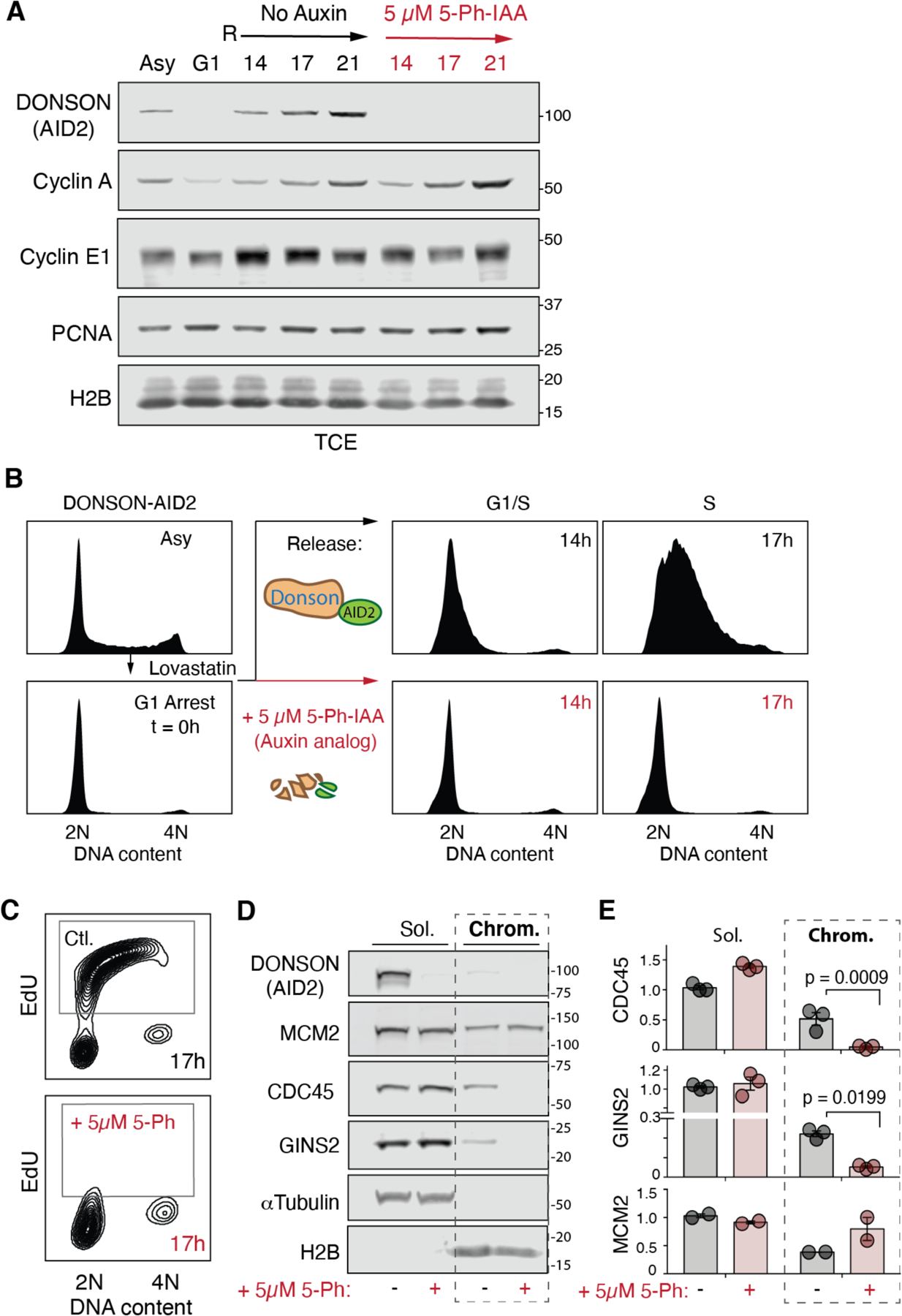
DONSON is required for DNA replication and CMG assembly in mammalian cells. **(A)** Immunoblot of synchronized DONSON-AID2 HCT116 cells. TCE, total cell extract; Asy, asynchronous; G1, G1-arrest; R, release, hours. **(B)** G1-synchronized cells fail to progress into S phase after 5-Ph-IAA depletion of DONSON. **(C)** DONSON depletion prevents DNA synthesis. EdU pulse-labeling at 17 hours post-release. Flow cytometry plots in panels (B) and (C), representative of *n* = 4 and *n* = 3 experiments, respectively, are quantified in figs. S16D and E, respectively. **(D, E).** DONSON is required for CDC45 and GINS recruitment to chromatin. (D) Immunoblots, soluble extract (Sol.) and chromatin-bound proteins (Chrom.) 17 hours post release. (E) Quantification, normalized to loading control (soluble, α-Tubulin; chromatin, Histone H2B) and wild-type protein levels. Datapoints, *n* = 3 experiments. Mean ± SEM.

DONSON is essential for cell proliferation in mammals (24). Therefore, to explore DONSON’s role in DNA replication, we generated a DONSON-AID2 degron HCT116 cell line that exhibits rapid degradation (≤ 1 hour) of endogenous DONSON upon addition of the auxin derivative, 5-Ph-IAA (fig. S16, A and B) (38). Acute DONSON depletion during ongoing replication impaired fork progression (fig. S16C), and DONSON was associated with replisomes following initiation in egg extracts (fig. S17), consistent with previous studies describing DONSON’s role in ongoing replication (23, 26). To test whether mammalian DONSON is also required for replication initiation, DONSON-AID2 cells were synchronized in G1 using lovastatin (39) and released into S phase. In the presence of 5-Ph-IAA to deplete DONSON (Fig. 5A), cells retained 2n DNA content, whereas in its absence, DNA content increased (Fig. 5B and fig. S16D). Furthermore, DONSON-depleted cells did not undergo detectable Ethynyl-2’-deoxyuridine (EdU) incorporation (Fig. 5C and fig. S16E). Despite the absence of replication, Cyclin A, Cyclin E, and CDK2 levels were unaffected by DONSON-depletion after release from the G1 arrest (Fig. 5A, fig. S16F), consistent with normal cell cycle progression. The parental HCT116 OsTIR^F74G^ cell line containing untagged DONSON was unaffected by 5-Ph-IAA (fig. S16G). In addition, chromatin recovery by cell fractionation showed that when cells were released from G1 in the absence of DONSON, GINS and CDC45 loading were substantially reduced (Fig. 5, D and E). In summary, our data show that DONSON is essential for CMG assembly in mammalian somatic cells, and they reinforce prior findings that DONSON promotes efficient replication fork progression.

### Defective CMG assembly is associated with microcephalic dwarfism in a mouse model

**Fig. 6.**
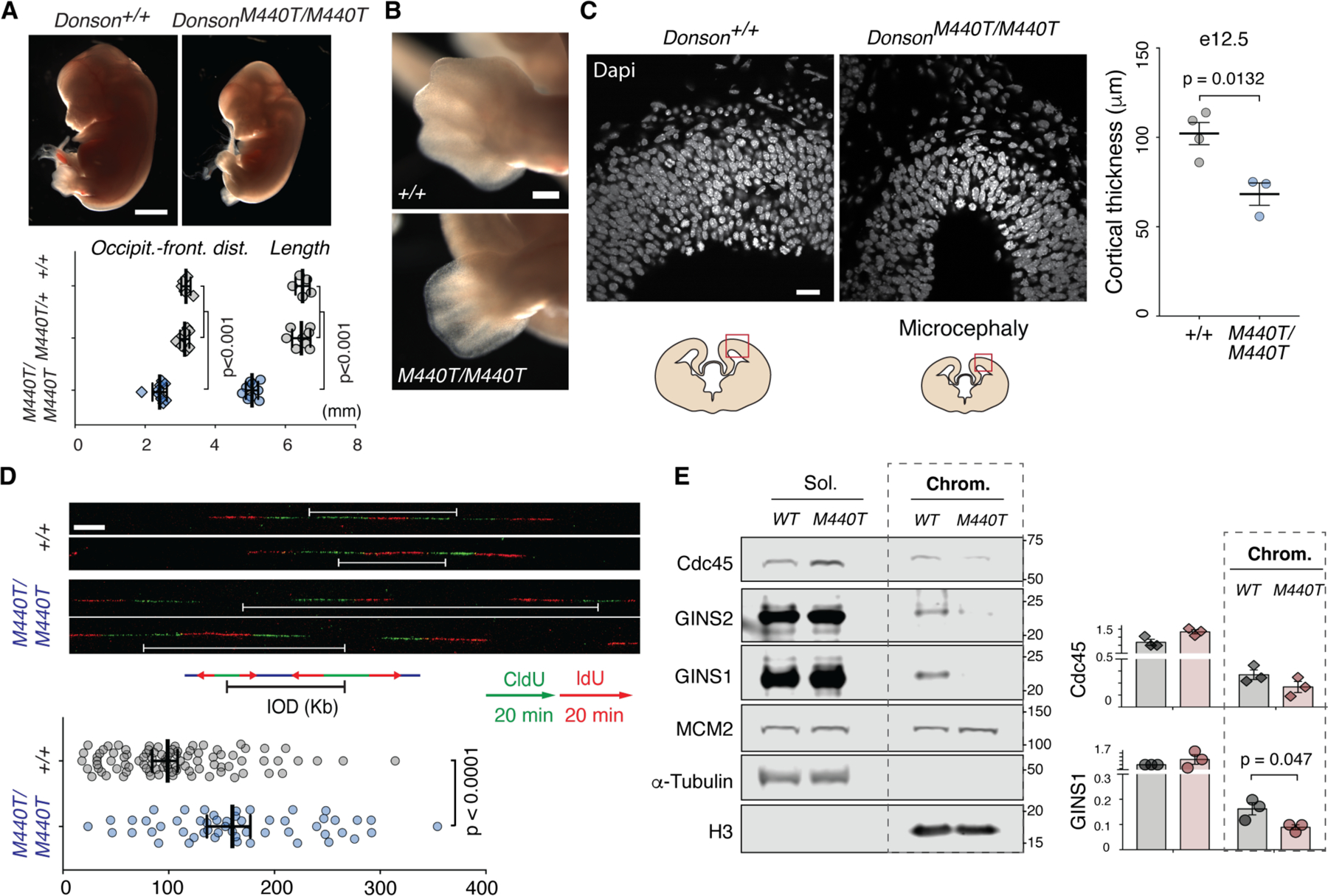
Homozygous *M440T* mutation impairs replication initiation and causes growth restriction and microcephaly in a mouse model. **(A-C)** *Donson*^*M440T/M440T*^ E13.5 mouse embryos exhibit growth restriction by mid-gestation with microcephaly and limb abnormalities. (A) Lateral view; scale bar 1 mm. Datapoints, individual mice, Mean ± SEM, *t*-test. Occipit.-front dist, occipital-frontal distance. (B) Oligodactyly in forelimb; scale bar 0.2 mm. See also fig. S18C. (C) Reduced cellularity as measured by cortical thickness is evident in the developing forebrain during neurogenesis (e12.5). Scale bar 20 µm. Measurements at dorsal-most point of telencephalon; datapoints, individual mice; Mean ± SEM, *t*-test. **(D)** Increased interorigin distance (IOD) in *Donson*^*M440T/M440T*^ cells. Representative images of dU-analog pulse-labeled DNA fibers. White brackets, measured IODs, mESCs. Data points plotted, fibers, pooled from *n* = 2 combing experiments. Kb, kilobases. Median ± 95% confidence interval; U-test. 91 wild-type and 53 *M440T/M440T* fibers were scored for IODs. **(E)** Reduced chromatin-associated GINS and Cdc45 indicates impaired CMG assembly in *Donson*^*M440T/M440T*^ mESCs. (Left) cell fractionation immunoblot. (Right) quantification. *n* = 3 experiments, mean ± SEM, *t*-test; normalization as in Fig. 5E.

Biallelic mutations that cause microcephalic dwarfism are clustered in the globular domain of DONSON (fig. S18A) and result in partial loss of function by reducing protein levels (23). Although most microcephalic dwarfism cases are compound heterozygotes, several homozygous mutations have been identified that are more easily modelled in isogenic systems. One such variant, M446T (23), was introduced into mice (at the corresponding location *M440*) through CRISPR/Cas9 genome editing (fig. S18B). The resulting *M440T/M440T* mouse exhibited microcephaly, reduced body size, and decreased limb length, confirming pathogenicity of the mutation at the organismal level and recapitulating a severe form of the human phenotype (Fig. 6, A to C, and fig. S18C). As previously reported in patient-derived cells (23), DONSON protein levels were substantially reduced in *Donson*^*M440T/M440T*^ mouse embryonic stem cells (mESCs; fig. S19A); DNA combing demonstrated fork asymmetry (fig. S19B), and ATR-mediated checkpoint signaling was attenuated (fig S19C). Additional analysis demonstrated that in both *M440T/M440T* mESCs and mouse embryonic fibroblasts (MEFs), interorigin distance was substantially increased (Fig. 6D and fig. S19D), whereas fork velocity (fig. S19E) and cell proliferation (fig. S19F) were reduced, consistent with a deficit in functional replisomes. Furthermore, the *M440T* mutation decreased chromatin-bound CDC45 and GINS levels in mESCs (Fig. 6E). These replication initiation phenotypes were not due to defective checkpoint signaling because inhibiting ATR has the opposite effect of DONSON deficiency in that it stimulates initiation events (origin firing) (40) and enhances CDC45 and GINS recruitment to chromatin (fig. S19G). Hence, in a murine model, defective CMG assembly induced by a DONSON patient-derived mutation is associated with microcephalic dwarfism.

### *Ab initio* prediction of protein function using AlphaFold-Multimer

Our initial screen for DONSON interactors was limited to the 70 replisome proteins because we knew from biochemical experiments that DONSON is required for CMG assembly (table S1). To assess whether *in silico* screening alone would point towards DONSON’s function in CMG assembly, we used AF-M to assess DONSON’s potential interaction with nearly all 20,000 known human proteins. As shown in table S2, TOPBP1, MCM3, POLE2, SLD5, and DONSON were among the 350 most confident DONSON interactors (sheet 2) but GO-term analysis did not identify DNA replication as a DONSON-associated function. However, when we performed a second round of structure predictions, taking into account that SLD5 is part of the tetrameric GINS complex and that TOPBP1 binds at the DONSON dimer interface (apparent from the first round of predictions; figs. S3 and S4), all five CMG assembly factors were among the 28 most confident, proteome-wide interactors (table S2, sheet 3). Alternatively, when we considered only the ~500 proteins associated with DONSON in the STRING database (41), CMG assembly factors were among the top 21 most confident interactors (table S2, sheet 4). These results suggest that *in silico* screening has potential as a general, *ab initio* approach to identify relevant interactors and thereby elucidate protein function.

## Discussion

Our data support a model in which DONSON scaffolds formation of a large pre-LC that delivers GINS to origins for CMG assembly (fig. S20). The predicted docking of DONSON onto MCM3 places GINS close to its binding site on CMG, suggesting that DONSON functions not only as a pre-LC scaffold but also as a molecular matchmaker. Mutations designed to disrupt the DONSON-MCM3 interaction impaired DONSON binding to chromatin, CMG assembly, and DNA replication. However, the pre-LC did not co-IP with MCM2-7 in nonreplicating extract, suggesting that the MCM3-DONSON interaction is context-dependent. Thus, we speculate that the pre-LC is first recruited to MCM double hexamers through phospho-dependent binding of TOPBP1’s BRCT0-2 repeats to TRESLIN (fig. S20A), followed by DONSON docking onto MCM3 (fig. S20, B and C, pink and blue circles). Because we have not been able to detect a direct physical interaction between DONSON and MCM3, the conclusion that the pre-LC docks onto MCM3 remains tentative. DONSON depletion disrupted not only GINS but also CDC45 recruitment to origins whereas TRESLIN and MTBP recruitment were unaffected. Given that CDC45 is known to bind MCMs weakly in the absence of GINS (5, 6), we favor the idea that CDC45 associates with origins normally in the absence of DONSON but dissociates during chromatin isolation due to the lack of full CMG assembly. We showed that CDC45 and GINS recruitment to chromatin were also defective in DONSON-deficient mammalian cells. Together with structure predictions in different organisms (table S1), evidence for DONSON-dependent GINS and CDC45 recruitment to chromatin in nuclear assembly egg extracts (42, 43), and data from worms (44), our evidence suggests that DONSON is generally required for CMG assembly in metazoans.

Our results also shed light on the role of Pol ε in replication initiation. A DONSON mutant (N67A) that disrupts Pol ε retention in the pre-LC has little effect on CMG assembly or replication efficiency, consistent with previous results that Pol ε is not required for CMG formation (19, 20). Notably, TOPBP1’s BRCT4-5 domain binds GINS on the same site that is occupied by POLE2 in the replisome (36) (fig. S9D), and Pol ε binds DONSON independently of GINS and TOPBP1, as shown by the DONSON^N67A^ mutant. Therefore, we propose that in the pre-LC, TOPBP1 uses its GINI and BRCT4-5 domains to occupy GINS, whereas Pol ε is flexibly attached, binding primarily to DONSON’s N-terminal disordered region (fig. S20A); after pre-LC docking and CMG assembly, Pol ε binds cooperatively to GINS and MCM2-7, displacing TOPBP1’s BRCT4-5 domains from GINS, which causes TOPBP1 dissociation from CMG (fig. S20, C and D).

In yeast, CDK-phosphorylation of Sld2 promotes Sld2 binding to Dpb11, which underlies pre-LC assembly. However, the closest vertebrate Sld2 homolog, RECQL4, functions downstream of CMG assembly (17, 18). Although DONSON and Sld2 share no sequence or structural homology and DONSON’s interaction with TOPBP1/Dpb11 does not appear to be regulated by phosphorylation, we propose that DONSON has replaced the function of Sld2 in vertebrate pre-LC assembly. DONSON is predicted to interact with Cyclin A and Cyclin E (table S1 and fig. S21), and DONSON depletion partially codepletes CDK2-Cyclin E, raising the possibility of a functional interplay between DONSON and CDKs that does not involve TOPBP1. Our evidence suggests a unified model in which CMG assembly involves a pre-LC in both yeast and metazoa but utilizes different architectures.

DONSON forms a dimer (Fig. 2F, fig. S3F and fig. S6), and dimerization does not clash with DONSON binding to other pre-LC components or MCM3 (fig. S4 and S5), suggesting that a dimeric pre-LC might dock onto MCM double hexamers (fig. S20A). However, given its predicted dimensions, the two MCM3 binding helices of the DONSON dimer would not be able to contact both MCM3s at the same time (fig. S20B, pink and black circles, and data S2). We speculate that disengagement and clockwise rotation of the two MCMs, as seen during CMG assembly in yeast (45), might enable DONSON dimer binding to both MCM3 molecules simultaneously (fig. S20C, pink and blue circles). Whether such a sequential CMG assembly mechanism occurs and whether it leads to concerted activation of sister replisomes is an important question for future studies.

Previous studies using siRNA and patient-derived cell lines implicated DONSON in ATR signaling, replication elongation, fork protection, and interstrand crosslink traversal (23, 26). In agreement, using degron-allele and isogenic mutant cell lines, we found that DONSON not only promotes CMG assembly but also affects downstream DNA replication events, including fork progression, fork stability, and checkpoint signaling. The interplay of these various functions is likely to be complex. Deficient checkpoint signaling may reflect a direct involvement of DONSON in ATR activation but could also be an indirect consequence of reduced origin firing. However, defective fork progression cannot be accounted for by DONSON’s role in replication initiation because reduced origin firing generally leads to faster fork rates as replication resources become more abundant (46). Indeed, knock-down of MTBP or TRESLIN, which act upstream of DONSON, reduces origin firing and increases fork rates, but unlike DONSON, does not cause fork asymmetry (11, 12, 47). Thus, DONSON’s phenotypes are consistent with a dual role in initiation and elongation. The mechanism by which DONSON acts downstream of replication initiation is unclear, especially given recent structural data (48), which indicates that Pol α binding to CMG would be incompatible with DONSON’s predicted interaction with CMG. One possibility is that if Pol α dissociates from CMG, DONSON binds, preventing GINS dissociation and/or regulating the replication stress response directly, similar to TOPBP1.

Meier-Gorlin syndrome (MGS), defined by growth restriction, microtia, and patella agenesis (49), is a form of microcephalic dwarfism specifically associated with genes encoding replication initiation factors (22). DONSON’s role in CMG assembly provides a mechanistic explanation for DONSON mutations discovered in MGS patients (50, 51). As replication licensing defects reduce cell proliferation during development (52), impaired CMG assembly could also limit embryonic cell divisions. This would reduce total cell number, resulting in the hypocellularity that underlies microcephalic dwarfism (21). Cell cycle progression is expected to be further impaired by slow fork progression observed when DONSON function is compromised. DONSON mutations are also associated with other microcephalic dwarfism disorders (microcephaly, short stature, and limb abnormalities, and microcephaly-micromelia syndrome), where brain size is disproportionately affected, and limb reduction abnormalities are evident (23, 24). These conditions may represent more severe forms of the same phenotypic spectrum; alternatively, they might reflect the disruption of other DONSON functions (23, 26).

Our results illustrate the power of *in silico* protein-protein interaction (PPI) screening. In a focused screen of DNA replication factors, AF-M clearly identified CMG assembly as the most likely DONSON function. Even when DONSON was screened against the entire human proteome, CMG assembly emerged as a probable DONSON function, especially when select hits were subjected to a second round of predictions guided by a knowledge of their quaternary structure. A much less computationally intensive approach that also identified DONSON’s functional partners involved AF-M screening of the ~500 proteins associated with DONSON in the STRING database. More proteins will have to be analyzed to develop robust and general strategies that successfully leverage structure prediction for *ab initio* discovery of protein function. Nevertheless, our results illustrate that, when combined with careful experimental validation, *in silico* PPI screening has great potential to accelerate mechanistic discovery.

## Materials and Methods

### Xenopus egg extracts and in vitro DNA replication

Experiments involving adult female (Nasco Cat #LM0053MX) *Xenopus laevis* performed at Harvard Medical School were approved by the Harvard Medical Area Standing Committee on Animals (HMA IACUC Study ID IS00000051-6, approved 10/23/2020, and IS00000051-9, pending approval). The institution has an approved Animal Welfare Assurance (D16-00270) from the NIH Office of Laboratory Animal Welfare.

*Xenopus* egg extracts were prepared as described previously (53). To execute *in vitro* DNA replication, the high-speed supernatant (HSS) of total egg lysate was first incubated with 15 ng of pBlueScript II per μL of HSS (30 ng to study binding of DONSON to chromatin, Figs. 1F and 4E, and fig. S17) for 30 mins at room temperature to promote replication licensing. Optionally, to inhibit licensing, HSS was supplemented with 0.4 μM of recombinant His-Geminin and incubated for 10 mins at room temperature before addition of plasmids. Replication was then initiated by adding two volumes of Nucleoplasmic Extract (NPE) supplemented with 1.93 mM DTT, 1.8 mM ATP, 18 mM phosphocreatine, and 4.5 μg/mL creatine phosphokinase. Where indicated, NPE was supplemented with 50 μg/mL recombinant GST-p27^Kip^ (“CDKi”) or 50 μM PHA-767491 (Sigma-Aldrich PZ0178, “DDKi”) and pre-incubated for 15 mins at room temperature before addition to HSS to inhibit CMG assembly.

### Analysis of total *in vitro* DNA synthesis

To monitor overall DNA synthesis, *in vitro* DNA replication reactions were supplemented with 0.16 μCi/μL of [α−^32^P]dATP (Perkin Elmer BLU512H500UC). At the indicated times after initiating replication by NPE addition, samples of the replication reactions were quenched in 5 volumes of replication stop buffer (80 mM Tris-HCl pH 8.0, 8 mM EDTA, 0.13% phosphoric acid, 10% Ficoll 400, 5% SDS, 0.2% bromophenol blue) supplemented with 20 μg of proteinase K (Roche 3115879001). The samples were incubated at 37°C for an hour to digest all proteins.

The samples were then separated by native agarose gel electrophoresis, using 0.9% agarose gels and 1× TBE buffer (89 mM Tris, 89 mM Boric acid, 2 mM EDTA pH 8.0). The gels were then surrounded by a positively charged membrane (GE/Cytiva Hybond-XL or Roche 11417240001) to prevent loss of nucleic acids, and dried. The dried gels were exposed to phosphor screens and imaged on the Typhoon FLA 700 PhosphorImager (GE Healthcare). Total DNA synthesis was determined by quantifying the total intensity of each lane using ImageJ.

### Expression and purification of recombinant *Xenopus* DONSON and *Xenopus* TOPBP1^1−530^

Untagged DONSON, FLAG-DONSON, and HA-TOPBP1^1−530^ were cloned into pGEX-6P1 vectors with sequences encoding a GST-tag and 3C protease cleavage site on the N-terminus. Indicated mutations were introduced using a Q5 Site-Directed Mutagenesis Kit (NEB #E0554S) and primers described in table S3. The vectors were then transformed into Rosetta (DE3) pLysS cells. For each purification, 1L of LB media was inoculated with the respective strain and grown to exponential phase at 37°C (OD_600_ ~0.4–0.8). Protein expression was induced with 1 mM IPTG for 16–18 hours at 16°C.

The cells were then harvested and resuspended in lysis buffer (50 mM HEPES-KOH pH 7.7, 500 mM NaCl, 5% glycerol, 5 mM DTT) supplemented with 1× cOmplete EDTA-free protease inhibitor cocktail (Roche 5056489001) and 200 μg/mL lysozyme. Cells were lysed by sonication and cleared by centrifugation in a Ti-45 rotor (Beckman Coulter) at 30,000 rpm for 1 hour at 4°C. The supernatant was collected, filtered using a 0.45 μm filter (Merck-Millipore SLHVR33RS) and incubated with 2 mL of Glutathione Sepharose 4B resin (GE/Cytiva 17075605) for 1 hour at 4°C. The resin was washed with 40 column volumes of lysis buffer, followed by 40 column volumes of wash buffer (50 mM HEPES-KOH pH 7.7, 150 mM NaCl, 5% glycerol, 5 mM DTT). The GST tag was cleaved by incubating the resin overnight at 4°C with 800 μg of PreScission Protease (fusion of GST and 3C protease). The flowthrough was collected, concentrated using an Amicon Ultra 10,000 MWCO centrifugal filter device (Millipore), cleared by centrifugation at 10,000 × *g* for 10 mins and subjected to size exclusion chromatography using a Superdex 200 Increase 10/300 GL column (GE Healthcare) and buffer containing 50 mM HEPES-KOH pH 7.7, 300 mM NaCl, 5% glycerol, 5 mM DTT. The appropriate fractions were collected and pooled, then concentrated using an Amicon Ultra 10,000 MWCO centrifugal filter device (Millipore). The concentrations of the purified proteins were quantified using at least three measurements on a NanoDrop One^C^ (ThermoFisher Scientific). Finally, the purified proteins were aliquoted, snap frozen in liquid N2, and stored at −80°C.

### Purification of recombinant *Xenopus* GINS

Recombinant GINS used in fig. S11B was the same preparation used and described previously (54).

Recombinant GINS used in fig. S13 was generated using the Acembl/MultiCol system (55) by first cloning the PSF1, PSF2 and SLD5 subunits of *Xenopus laevis* GINS into a pDC donor plasmid and PSF3 (with a C-terminal His6 tag connected through a LPETG tag and 10-aa linker) into a pACE2 acceptor plasmid. Cre-recombination was then used to assemble all subunit of GINS into a single expression plasmid. The H76A mutation in SLD5 was introduced into the pDC donor plasmid prior to Cre-recombination using a Q5 Site-Directed Mutagenesis Kit (NEB #E0554S) and primers described in table S3.

The vectors were then transformed into Rosetta (DE3) pLysS cells. For each purification, 1L of LB media was inoculated with the respective strain and grown to exponential phase at 37°C (OD_600_ ~0.4–0.8). Protein expression was induced with 1 mM IPTG for 4 hours at 30°C. Cells were then harvested and resuspended in lysis buffer (20 mM Tris-HCl pH 8.0, 500 mM NaCl, 20 mM Imidazole, 1 mM PMSF, 1mM DTT, 5% glycerol) supplemented with 2× cOmplete EDTA-free protease inhibitor cocktail (Roche 5056489001) and 2 mg/mL lysozyme. Cells were lysed by sonication and cleared by centrifugation in a Ti-45 rotor (Beckman Coulter) at 30,000 rpm for 1 hour at 4°C. The supernatant was collected, filtered using a 0.45 μm filter (Merck-Millipore SLHVR33RS) and incubated with 1 mL of Ni-NTA Superflow resin (Qiagen) for 1 hour at 4^o^C. The resin was washed with 100 column volumes of lysis buffer followed by 5 column volumes of elution buffer (20 mM Tris-HCl pH 8.0, 500 mM NaCl, 250 mM Imidazole, 1 mM PMSF, 1mM DTT, 5% glycerol). The eluate was diluted to 150 mM NaCl using MonoQ buffer (20 mM Tris-HCl pH 7.5, 1 mM DTT, 5% glycerol) and subjected to anion exchange chromatography using a MonoQ 5/50 GL column (Cytiva) with a 150–700 mM NaCl gradient in MonoQ buffer. GINS eluted at 350 mM NaCl. The appropriate fractions were collected and pooled, then de-salted to 150 mM NaCl using a PD10 de-salting column and concentrated using an Amicon Ultra 10,000 MWCO centrifugal filter device (Millipore). The concentrations of recombinant GINS from bacterial expression were determined by running aliquots alongside a titration of recombinant GINS from Sf9 expression (54) on a 4–15% Mini-PROTEAN TGX Precast Protein Gel stained using InstantBlue stain (Novus ISB1L). Total lane intensity was quantified using ImageJ. The purified proteins were aliquoted, snap frozen in liquid N2 and stored at −80°C.

### Expression of proteins in wheat germ protein expression system

FLAG-DONSON and HA-TOPBP1 (various constructs) were cloned into pF3A WG (BYDV) Flexi vectors. Indicated mutations were introduced using a Q5 Site-Directed Mutagenesis Kit (NEB #E0554S) and primers described in table S3. Plasmids were maintained in DH5α cells and purified using QIAprep Spin Miniprep Kits (Qiagen). The proteins were expressed in the TnT^®^ SP6 High-Yield Wheat Germ Protein Expression System (Promega) by mixing 3 volumes of the extract with 2 volumes of 100 ng/μL purified plasmid and incubating at 25°C for 2 hours. Extracts containing expressed proteins were used immediately.

### Immunodepletions and rescue experiments

For immunodepletion of endogenous DONSON, we raised a rabbit polyclonal antibody against a peptide comprising amino acids 11–23 of *Xenopus* DONSON (Biosynth project #4616). 0.3 volumes of the 1 mg/mL antibody were incubated with 1 volume of Dynabeads Protein A (Invitrogen 10002D) by gently rotating at 4°C overnight. 1.5 volumes of extract was immunodepleted by three rounds of incubation with 1 volume of antibody-charged Dynabeads, by gently rotating at 4°C for 1 hour per round.

For immunodepletion of endogenous TOPBP1, we raised a rabbit polyclonal antibody against a peptide comprising amino acids 498–510 of *Xenopus* TOPBP1 (Biosynth project #5620). 3 volumes of the 1 mg/mL antibody was incubated with 1 volume of Protein A Sepharose Fast Flow antibody purification resin (GE/Cytiva #17127903) by gently rotating at 4°C overnight. 5 volumes of extract was immunodepleted by three rounds of incubation with 1 volume of antibody-charged Protein A Sepharose beads, by gently rotating at 4°C for 1 hour per round.

For immunodepletion of endogenous GINS, anti-GINS antibodies (Pocono #34300), affinity-purified as previously described (54), were used. 5 volumes of the 1 mg/mL antibody was incubated with 1 volume Protein A Sepharose Fast Flow antibody purification resin (GE/Cytiva #17127903) by gently rotating at 4°C overnight. 5 volumes of NPE was immunodepleted by three rounds of incubation with 1 volume of antibody charged Protein A Sepharose beads, by gently rotating at 4^o^C for 1 hour per round. 5 volumes of HSS was immunodepleted by two rounds of incubation under the same conditions.

In DONSON rescue experiments (all except fig. S1, B and C), NPE was supplemented with 0.3 μM final concentration of recombinant human CDK2-Cyclin E1 (ProQinase #0050-0055-1) and incubated for 15 mins at room temperature before initiating replication, to compensate for the co-depletion of endogenous CDK2-Cyclin E during the immunodepletion of endogenous DONSON. Recombinant DONSON (WT and mutants) was added at a final concentration of 150 nM in NPE and incubated for 15 mins at room temperature before initiating replication.

In GINS rescue experiments (fig. S13) recombinant GINS was added at a final concentration of either 180 nM or 270 nM in NPE and incubated for 15 minutes at room temperature before initiating replication.

For rescues using proteins expressed in wheat germ extract (figs. S12, S14, and S15), 1 volume of the appropriate wheat germ extract was added to 4 volumes of NPE and incubated for 15 mins at room temperature before initiating replication.

### SDS-PAGE and immunoblotting of samples from *Xenopus* egg extract experiments

All samples to be analyzed were boiled in Laemmli buffer (50 mM Tris-HCl pH 6.8, 2% SDS, 10% glycerol, 0.1% bromophenol blue, 5% β-mercaptoethanol). Unless stated otherwise, samples were run in 4 to 15% Mini-PROTEAN TGX Precast Protein Gels (Bio-Rad #4561086) or 4 to 15% Criterion TGX Precast Midi Protein Gels (Bio-Rad #5671085) using Tris-Glycine-SDS Running Buffer (25 mM Tris-HCl pH 8.3, 192 mM glycine, 0.1 % SDS). The samples were run alongside EZ-Run Prestained Rec Protein Ladder (Fisher BioReagents #BP36031) to infer the size of the protein bands.

For Coomassie staining, gels were stained using InstantBlue stain (Novus ISB1L) for at least 1 hour at room temperature. For immunoblotting, gels were transferred to PVDF membranes (Thermo Scientific #88518) in transfer buffer (25 mM Tris pH 8.5, 192 mM glycine, 20% methanol). The membranes were blocked in 1× PBST containing 5% (w/v) nonfat milk for 30 mins at room temperature with gentle shaking, and incubated with primary antibodies diluted in 1× PBST containing 1% (w/v) BSA overnight at 4°C with gentle shaking. Membranes were then washed extensively with 1× PBST and incubated with secondary antibodies diluted in 1× PBST containing 5% (w/v) nonfat milk for 1 hour at room temperature with gentle shaking. Membranes were washed again extensively with 1× PBST, developed using ProSignal Pico ECL Spray (Prometheus Protein Biology Products #20-300S) or SuperSignal West Dura extended duration substrate (Thermo Scientific 34075) and imaged using an Amersham Imager 600 (GE Healthcare).

Rabbit polyclonal antibodies against the following proteins were used as primary antibodies for western blotting:

DONSON (1:5000, described above)

MCM7 [1:12,000, (31)]

MCM4 (1:4000, Bethyl #A300-193A, RRID: AB_162720)

MCM3 [1:4000, Santa Cruz (H-215) #sc-292857]

CDC45 [1:20,000, (56)]

GINS [1:5000, (54))

POLEcat [1:5000, (57)]

POLA1 (1:5000, Pocono #35956 raised against the N-terminal 340aa fragment of *Xenopus laevis*

POLA1, used in Figs. 1E and 4E)

PCNA [1:5000, (58)]

TOPBP1^498−510^ (1:5000, described above and used in fig. S15)

FLAG [1:5000, (57)]

HA [1:1000, Cell Signaling (C29F3) #3724, RRID: AB_1549585]

Cyclin E [1:5000, (27)]

CDK2 [1:5000, (28)]

Histone H3 (1:500, Cell Signaling #9715, RRID: AB_331563).

Mouse monoclonal antibodies against the following proteins were used as primary antibodies for western blotting:

GST (1:3000, Cell Signaling #2624, RRID: AB_2189875)

Rabbit polyclonal antibodies against TRESLIN [1:1000, (8)], MTBP [1:500, (12)], and RECQL4 [1:1000, (17)] were generous gifts from William Dunphy (California Institute of Technology, USA).

Rabbit polyclonal antibodies against TOPBP1 [1:2500, (13), used in all TOPBP1 blots except in fig. S15] and POLA1 [1:5000, (59), used in all POLA1 blots except in Figs. 1E and 4E] were generous gifts from Matthew Michael (University of Southern California, USA).

A rabbit polyclonal antibody against POLE2 [1:7500, (19)] was a generous gift from Shou Waga (Japan Women’s University, Japan).

The following secondary antibodies were used:

Goat anti-rabbit horseradish peroxidase-conjugated (Jackson ImmunoResearch, 111-035-003, RRID: AB_2313567) at 1:10,000-1:30,000 dilution.

Light chain specific mouse anti-rabbit horseradish peroxidase-conjugated (Jackson ImmunoResearch, 211-032-171, RRID: AB_2339149) at 1:5000 dilution.

Rabbit anti-mouse horseradish peroxidase-conjugated (Jackson ImmunoResearch, 315-035-003, AB_2340061) at 1:2000 dilution.

### Plasmid pull-down (chromatin pull-down)

Plasmid pull-downs were performed essentially as described (60). Briefly, 1 volume of streptavidin-coated magnetic beads (Dynabeads M-280, Invitrogen 11206D) was incubated with 6 volumes of binding buffer (50 mM Tris-HCl pH 7.5, 150 mM NaCl, 1 mM EDTA, 0.02% Tween 20) containing 0.2 μM of biotinylated recombinant LacI for 40 mins at room temperature. The beads were then washed thrice with stop buffer (20 mM HEPES-KOH pH 7.7, 100 mM KCl, 5 mM MgCl_2_, 0.5 M sucrose, 0.25 mg/mL BSA, 0.03% Tween 20), and resuspended in 5 volumes of the same buffer. The washed beads were then aliquoted and chilled on ice.

At indicated times after initiating replication by NPE addition, samples of replication reactions were added to the bead aliquots at a 1-to-10 ratio and gently rotated for 30 mins at 4°C. The beads were then washed thrice with wash buffer (20 mM HEPES-KOH pH 7.7, 100 mM KCl, 5 mM MgCl_2_, 0.25 mg/mL BSA, 0.03% Tween 20). Bound proteins were eluted by boiling with 1× Laemmli buffer and subjected to analysis by SDS-PAGE and immunoblotting. For all figures except Figs. 1F, 4E and fig. S17, bound proteins eluted from 6 ng of plasmids were loaded in each well. For Figs. 1F, 4E and fig. S17, bound proteins eluted from 36 ng of plasmids were loaded in each well. To infer the efficiency of the plasmid pull-downs, an equivalent of 5% of the replication reaction subjected to plasmid pull-down (“input”) was loaded on the gels alongside the plasmid pull-down samples.

### Immunoprecipitation

For anti-FLAG immunoprecipitations, Anti-FLAG M2 Magnetic Beads (Millipore M8823) were washed thrice in IP wash buffer (10 mM HEPES-KOH pH 7.7, 50 mM KCl, 2.5 mM MgCl_2_, 250 mM sucrose, 0.1 mg/mL BSA, 0.02% Tween 20) and used in aliquots containing 2 μL of packed beads. The beads were optionally pre-immobilized with proteins expressed in wheat germ extract (Fig. 4D and figs. S12 and S14) by incubating each aliquot of beads with 20 μL of wheat germ extract expressing the desired FLAG-tagged protein for 1 hour at 4°C with gentle rotation. Each aliquot of beads was incubated with 15 μL of 30% NPE (diluted in IP wash buffer) containing 1 μM of recombinant FLAG-DONSON (omitted if beads were pre-immobilized with protein expressed in wheat germ extract), for 1 hour at 4°C with gentle rotation. The beads were then washed thrice with cold IP wash buffer. To elute bound proteins, each aliquot of beads was incubated with 15 μL of IP wash buffer containing 1 mg/mL of 3× FLAG peptide (Sigma-Aldrich F4799) for 1 hour at room temperature with gentle rotation.

For immunoprecipitation of mixed purified proteins (figs. S11B and S13C), each purified protein was added at 0.5 μM to IP wash buffer and incubated at room temperature for 15 mins. These mixtures were incubated with the beads instead of NPE, and for 30 minutes at room temperature instead of 1 hour at 4°C. Otherwise, the immunoprecipitation was performed essentially as described above.

For anti-HA immunoprecipitations (figs. S10 and S15), Anti-HA Magnetic Beads (Pierce 88836) were washed thrice in IP wash buffer and used in aliquots containing 0.1 mg of beads (10 μL of bead slurry). The beads were pre-immobilized with HA-TOPBP1 proteins expressed in wheat germ extract by incubating each aliquot of beads with 18 μL of wheat germ extract expressing the desired protein for 1 hour at 4°C with gentle rotation. Each aliquot of beads was then incubated with 15 μL of 30% NPE (diluted in IP wash buffer) for 1 hour at 4°C with gentle rotation. The beads were then washed thrice with cold IP wash buffer. Bound proteins were eluted by boiling each aliquot of beads with 30 μL of 1× Laemmli buffer.

For immunoprecipitation of endogenous PSF3 (Fig. 3B), 0.3 volumes of 1 mg/mL anti-PSF3 antibody (Bethyl 61582A) was incubated with 1 volume of Dynabeads Protein A (Invitrogen 10002D) by gently rotating at 4°C overnight. The antibody was crosslinked to the beads using dimethyl pimelimidate (DMP) (Thermo Scientific 21666), then washed thrice in IP wash buffer and used in aliquots containing 0.3 mg of beads (10 μL of bead slurry). Each aliquot of beads was incubated with 15 μL of 30% NPE (diluted in IP wash buffer) for 1 hour at 4°C with gentle rotation. The beads were then washed thrice with cold IP wash buffer. Bound proteins were eluted by boiling each aliquot of beads with 30 μL of 1× Laemmli buffer.

6 μL of each eluate sample was loaded on each gel and analyzed by immunoblotting. To infer the efficiency of the immunoprecipitations, an equivalent amount of extract as that subjected to immunoprecipitation (“input”) was loaded on the gels alongside the eluate samples. In Fig. 3E, extracts were supplemented with 50 μg/mL recombinant GST-p27^Kip^ (“CDKi”) or 20 U/μL Lambda protein phosphatase (New England BioLabs P0753) and treated for 30 mins at room temperature prior to immunoprecipitation.

### Mass photometry

Wild-type recombinant FLAG-DONSON was analyzed on a Refeyn TwoMP mass photometer at the Harvard Medical School Center for Macromolecular Interactions. The mass photometer was calibrated with a protein calibration mix containing 10 nM β-amylase (Sigma Aldrich A8781) and 3 nM Thyroglobulin (Sigma-Aldrich 609310) prior to taking measurements (concentrations listed were the final concentrations in droplet). Recombinant wild-type FLAG-DONSON was diluted to 200 nM in egg lysis buffer (10 mM HEPES-KOH pH 7.7, 50 mM KCl, 2.5 mM MgCl_2_, 250 mM sucrose). For each measurement, the objective was focused using an 18 μL droplet of PBS, 2 μL of 200 nM FLAG-DONSON was mixed into the droplet, and sample data was collected immediately. Figures and Gaussian fits were generated using the Refeyn DiscoverMP software.

### AlphaFold2-multimer (AF-M) screen

To discover novel DONSON interactors within DNA replication pathways, we performed an *in silico* screen using the AF-M program developed by DeepMind (35, 61). We folded DONSON homologs pairwise against core corresponding replisome proteins from *Homo sapiens*, *Xenopus laevis*, *Drosophila melanogaster*, and *Caenorhabditis elegans*. See table S1 for proteins examined in each organism.

In all cases, we ran all 5 of the AF-M models for 3 recycles with version 3 weights, templates enabled, and no dropout. These runs were performed using a local installation of Colabfold v1.5 (62) running on a Linux server equipped with 40GB NVIDIA A100 GPUs. Multiple sequence alignments (MSAs) and template inputs to the AlphaFold network were generated within the Colabfold pipeline which routes protein sequences to another server running the MSA software MMseqs2 (63). All predictions were generated using a combination of the paired and unpaired MSAs supplied by MMseqs2. Except in one case (fig. S7), predicted structures were not relaxed.

To analyze the predictions produced by AlphaFold 2, we also established a separate analysis pipeline written in python. The analysis pipeline integrates spatial information about residues as well as confidence and accuracy metrics predicted by AlphaFold 2. The analysis iterates through each residue in a protein chain and analyzes its position and confidence relative to residues in other protein chains to find contacts. We defined a contact as a unique pair of residues that have an average predicted local distance difference threshold (pLDDT) > 50, a minimum predicted Alignment Error (pAE) < 15 angstroms, and 2 non-hydrogen atoms closer than 8 angstroms. For each prediction we defined an interface as the set of all “contacts” (residue pairs) between 2 amino acid chains. We generated a series of interface statistics such as average pAE and average pLDDT, by averaging the individual values of these statistics across all contacts. We additionally calculated the predicted DOCKQ (pDOCKQ) value for all predictions as an estimate of the interface accuracy with a score ranging from 0 (worst) to 1 (best) (64).

Once a list of contacts had been identified in each prediction, these residue pairs/contacts were compared across all predictions generated for a particular complex. This comparison then allowed us to calculate aggregate metrics that quantify how well each AlphaFold model’s predictions agree on an interface. The two primary metrics we calculated were the “average n models” and “max n models”. The “average n models” statistic represents the average number of models that predict each contact and is calculated by finding all unique contacts across all predictions, counting how many models predicted each of these contacts, and then averaging the result across all the unique contacts. This procedure also let us calculate the “max n models” which is the maximum number of models that predict a specific contact. In both cases, the numbers are bounded between 1 and the number of models run with higher values indicating higher levels of agreement between models/predictions. Because we always ran five models, these metrics range from 1 to 5. The data for our DONSON screen across the replisome proteins of 4 species is presented as Table S1. The code we used to analyze the predictions is available on Zenodo (65).

To perform a human proteome wide screen for potential DONSON interactors, we downloaded all the canonical isoform sequences for 20,424 Swiss-Prot reviewed human proteins from the UNIPROT web portal on 8 July, 2023. We removed any redundant amino acid sequences and any proteins that were shorter than 15 residues or longer than 3034 residues (to prevent GPU memory exhaustion). This left a set of 20,190 proteins representing 98.8% of the known human proteome. We ran all these proteins paired with DONSON for 3 recycles with version 3 weights, templates enabled, and no dropout using models 1,2, and 4 through the aforementioned Colabfold pipeline. The results were analyzed through the same python analysis script and are summarized in table S2 sheet 2, where each row represents 1 pair that was folded. These results were sorted by avg_n_models (desc), pdockq (desc), avg_interface_pae (asc) and assigned a rank. Because we ran 3 models/3 repeats for this set, the maximum/best achievable avg_n_models and max_n_models for pairs was 3.

To identify proteins previously associated with human DONSON, we queried the STRING database through its REST API on 19 July, 2023. We set no minimum limits for required scores and retrieved all available entries. This resulted in a list of 536 proteins that we mapped to 507 proteins from our proteome-wide screen. The results of this mapping were tabulated in sheet 4 of table S2, where proteins with a DONSON STRING association were assigned a value of 1 for the in_STRING_db column. All other proteins were assigned a value of 0. We re-sorted the table based on STRING association (STRING DB followed by AF metrics as described above). This sorting protocol resulted in new rankings that are presented in Sheet 4.

### AlphaFold-based modeling of protein structures in ChimeraX

Fig. S4A: The complex shown in fig. S4A was assembled stepwise, as follows. AF-M was used to first predict the structure of a *Xenopus* DONSON (“#1” in figure), GINS, and MCM3 complex (PAE plots shown in fig. S4B), and the resulting PDB file was opened in ChimeraX. Disordered DONSON residues (1–6, 26–154, 330–369) were deleted, leaving only the globular domain and GINS-binding peptide (residues 7–25). MCM3 residues 659–807 were also deleted. Separately, TOPBP1 and GINS were folded together using AF-M (PAE plots for the human prediction shown in fig. S9; prediction for *Xenopus* complex looks very similar, as shown in fig. S9C). All TOPBP1 residues except 475–492 (GINI peptide) and 538–734 (BRCT4-5) were deleted, and the resulting TOPBP1-GINS complex (GINS hidden) was aligned with the PSF1 subunit of the above DONSON-GINS-MCM3 complex. Separately, two copies of DONSON (residues 155–579) and 1 copy of the TOPBP1 BRCT3 domain (residues 343–447) were folded with AF-M (PAE plot shown in fig. S4D) and aligned to the DONSON-GINS-MCM3 complex, revealing the position of the second copy of DONSON (brick red), and demonstrating that the TOPBP1 BRCT3 domain binds the DONSON dimer interface. Finally, DONSON was folded with POLE2 (PAE plots shown in fig. S4C), all DONSON residues except those interacting with POLE2 (65*–*72) were deleted. The POLE2-DONSON complex was not aligned with the rest of the complex but instead is shown separately in fig. S4A.

Fig. S5A: Same as fig. S4A except all proteins were human, and the disordered residues deleted in DONSON were 1–6, 26–73, 83–154, 325–352. MCM3 residues 659–808 were deleted. The BRCT3 domain of TOPBP1 comprises residues 340–450.

Fig. 2H: Top, same as fig. S4A (left side) but the second copy of DONSON and MCM3 were hidden. Bottom, the structure shown in the top panel was docked onto the human cryo-EM replisome structure [PDB: 7PLO (66), all but MCM2-7 and CDC45 were hidden] using the common MCM3 subunit, and MCM3 from the AlphaFold structure was hidden.

Fig. 2I. Same as fig. S4A (right side) but the second copy of DONSON and MCM3 were hidden.

Fig. 3D and 4C: Same as fig. S4A (right side) except that MCM3 and the second copy of DONSON were hidden.

### RMSD Calculation (fig. S7)

The MCM3-DONSON-GINS complex predicted by AF-M was relaxed using AMBER (https://ambermd.org/index.php), and hydrogen atoms were removed with Coot (67). The structure was opened in ChimeraX together with PDB:7PLO, and the RMSD was calculated for MCM3 and the GINS complex between the two structures.

### Mammalian cell culture

All cells were grown at 37°C and 5% CO_2_, and shown to be mycoplasma negative through routine testing. HCT116 cells (human colonic cancer cell line) were grown in McCoy’s 5A Modified media (Gibco Cat. No. 26600023) supplemented with 10% FCS (Gibco Cat. No. 10270–106; lot 2078421) 1 x penicillin and 2 mM L-Glutamine. MEFs (derivation, see generation of knock-in mice section), were grown at 3% O_2_ in DMEM (Gibco Cat. 41965–039; lot 2340231) supplemented with 10% FCS (Gibco Cat. No. 10270–106; lot 2078421), 1 X penicillin/streptomycin, 0.1 µM 2-mercaptoethanol (Gibco Cat. No. 31350–010; lot 2328476). mESCs (*Mus musculus*, 129/Ola E14 parental cell line) were maintained in serum/LIF media containing G-MEM BHK-21 (Invitrogen Cat. No. 21710–025), 10% FCS (Gibco Cat. No. 10270–106; lot 2078421), 1X sodium pyruvate (Sigma Cat. No. S8636), 1X MEM Nonessential Amino Acid Solution (Sigma Cat. N. M7145) 1 x penicillin and 2 mM L-Glutamine, 1:500 serum-conditioned media containing LIF and 0.5 μM β-Mercaptoethanol (Gibco Cat. No. 21985–023), fed every day and split every two days. Where indicated, 1µM AZD6738 (AdooQ Bioscience Cat. No. A15794), ATR inhibitor (ATRi), and/or 2mM Hydroxyurea (HU) (Sigma Cat. No H8627) were added to culture media for the times specified in the relevant experiments.

### DONSON-AID2 cell line

To establish the HCT116 DONSON-AID2 cells, a parental cell line expressing OsTIR1(F74G) was transfected with two CRISPR plasmids targeting the C-terminal coding region of the *DONSON* gene (targets: 5’-TTAGGCTTACTTTGGTGTTC-3’, 5’-TTAGGCTTACTTTGATGTTC-3’) and a donor plasmid encoding mAID-mClover and a hygromycin resistant marker following a published protocol (38, 68). After selecting clones in the presence of hygromycin (100 µg/mL), bi-allelic insertion was confirmed by genomic PCR. Subsequently, expression of DONSON-mAID-mClover protein was confirmed by western blotting.

### Cell synchronization of HCT116 cells

Synchronization was performed as previously described (69). In brief, 1.0 to 1.5 × 10^5^ cells were seeded into 6-well plates and grown for 1 to 2 days until 50% confluent. G1 arrest was induced by 24 to 30 h treatment with 20 µM Lovastatin (Thermo Scientific Cat. No. 15590584). G1-arrested cells were washed once with lovastatin-free medium and grown in medium containing 2 mM DL-Mevalonolactone (Sigma- Aldrich, M4667) with/out 5 μM 5-phenyl-1H-indole-3-acetic acid (5-Ph-IAA; MedChemExpress Cat No. HY-134653).

### Immunoblotting in mammalian cell experiments

Total cell extracts were prepared in urea lysis buffer containing 8 M urea, 50 mM Tris-HCl, pH 7.5, 150 mM β-mercaptoethanol, protease inhibitors and PhoSTOP (Roche Cat. No. 04693132001 and 4906837001). Lysed samples were sonicated 7 × 30 sec ON/OFF cycles using a Bioruptor (Diagenode). Protein electrophoresis was performed using NuPAGE 10% or 4 to 12% Bis-Tris mini protein gels (Invitrogen Cat. No. NP0336BOX, NP0301BOX) and MOPS running buffer (Cat. No. NP0001) at 80 to 130 V. Wet transfer of proteins to Immobilon-FL PVDF membrane (Millipore Cat. No. IPFL00010) was performed at 100 V for 60 to 75 min at 4°C. After transfer, membranes were washed in methanol, air-dried and re-activated in methanol, washed in 1X Tris-buffered saline/0.2% Tween-20 (Sigma. Cat. No. P1379) (TBS-T), and blocked in TBS-T/2.5% BSA (Roche Cat. No. 10735086001, lot 64758420) for 1 h at room-temperature (RT). Blots were incubated overnight (O/N) in TBS-T/2.5% BSA containing primary antibody. After 4 × 5 min washes in TBS-T, blots were incubated with secondary antibodies (1:20,000 to 30,000) for 1 hour at RT, washed 4 × 5 min in TBS-T and rinsed in TBS before acquisition using a LI-COR Odissey^®^ CLx imager. ImageStudio software was used for quantification.

The following primary antibodies were used for immunoblotting mammalian samples:

Mouse anti-Histone H2B, clone 5HH2-2A8 (Millipore Cat. No. 05–1352), lot 3836574, 1:20,000. RRID:AB_10807688

Mouse anti-Alpha tubulin (α-Tub), clone B-5-1-2 (Sigma Cat. No. T6074), lot 037M4804V, 1:10,000. RRID:AB_477582

Mouse anti GINS1 (Millipore Cat. No. MABE2033), lot Q3876194 1:1500

Rabbit anti-GINS2/Psf2 (Atlas Cat. No. HPA057285, lot A113986): 1:2500–10,000. RRID:AB_2683398

Rabbit anti-CDC45 (D7G6; CST Cat. No. 11881S), lot 1; 1:1500. RRID:AB_2715569

Rabbit anti-pChk1-ser317 (CST Cat. No. 2344, 1:2500). RRID:AB_331488

Mouse anti-Chk1 (G-4; Santa Cruz Cat. No. sc-8408, 1:2000). RRID:AB_627257

Rabbit anti-CDK2 (CST Cat. No 2546). RRID:AB_2276129

Rabbit anti-Cyclin E1 (Proteintech Cat. No. 11554-1-AP). RRID:AB_2071066

Rabbit anti-hDONSON and mDonson (generated in authors’ lab); 1:2000 and 1:750 respectively.

The following secondary antibodies were used for immunoblotting mammalian samples:

IRDye 680RD Goat anti-Rabbit IgG (H + L) Highly Cross-Adsorbed, 0.1 mg (925–68071). RRID:AB_2721181

IRDye 800CW Goat anti-Mouse IgG (H + L) Highly Cross-Adsorbed, 0.1 mg (925–32210). RRID:AB_2687825

IRDye 680RD Goat anti-Mouse IgG (H + L) Highly Cross-Adsorbed, 0.1 mg (925–68070). RRID:AB_2651128

IRDye 800CW Goat anti-Rabbit IgG (H + L) Highly Cross-Adsorbed, 0.1 mg (925–32211). RRID:AB_2651127

### Generation of DONSON antibodies used for mammalian cell experiments

A rabbit polyclonal antibody against human DONSON (hDONSON) was generated previously (23), raised against amino acid residues 1–125 of human DONSON, purified after expression in *E. coli* from the pGEX-6P-1 expression vector. Polyclonal antibody against mouse Donson was also raised in rabbits using full-length mouse Donson (mDonson), purified after expression in *E. coli* from a pET28a-His-SUMO expression vector. Antibodies were affinity-purified from rabbit sera (Eurogentech) and specificity was established using lysates from siRNA-transfected cells, patient cells and knock-in mESCs.

### Mammalian cell fractionation

Soluble and chromatin samples prepared using CSK buffer (70) (10 mM PIPES, pH 6.8, 300 mM Sucrose, 100 mM NaCl, 1.5 mM MgCl_2_, 0.5% Triton-X-100, 1 mM ATP (VWR Cat. N. R0441), 1 mM DTT and 0.2 mM PMSF plus 1X protease inhibitors and PhoSTOP). Harvested cells were washed in cold phosphate-buffered saline (PBS), and resuspended in 500 µl CSK for 10 min on ice, then centrifuged at 1600 g for 6 min, with supernatant collected as soluble fraction. Pellets were washed twice by resuspending in 0.5 and 1 ml CSK buffer for 5 min, and centrifuged at 1,600 g. Chromatin fractions were obtained by resuspending the pellet in 500 µl 2X Sample loading buffer (2X SLB: 100 mM Tris–HCl (pH 6.8), 4% SDS, 20% Glycerol, 0.2% Bromophenol blue, 10% β-Mercaptoethanol) and sonicated 15 × 30 sec ON/OFF cycles on a Bioruptor. 100 µl 6X SLB was added to the soluble fraction, and then equal volumes of soluble and chromatin fractions loaded for SDS-PAGE and immunoblotting as outlined above.

### Flow cytometry

Cells harvested for each time point from a well of a 6-well plate were pelleted at 1200g, resuspended in 75 µl PBS and fixed by adding 1 ml 100% freezer-cold ethanol with gentle vortexing and stored at –20°C. Fixed cells were pelleted by centrifugation at 1300g for 5 min, washed in 1X PBS/0.1% Triton-100-X (PBS-T) and resuspended in 1 ml of 2 µg/ml DAPI/PBS-T and incubated for 1 to 16 h at 4°C. For DNA content determination, cells were pelleted at 1200g and resuspended in 350 µl PBS for analysis on a Cytoflex S analyzer (Beckman Coulter), Violet 405 nm laser, 450/45 bandpass filters, 20,000 events in the single-cell population gate recorded. Data analysis performed using Flowjo v10.8.1 (Flowjo LLC, BD), with G1, S, G2/M fractions quantified using the Dean-Jett-Fox model with G1 and G2 peaks constrained based on histogram of asynchronous cells. To assess EdU incorporation, cells were pulse labelled for 15 min with 40 µM Ethynyl-2’-deoxyuridine (EdU; Sigma Cat. No. 900584-50mg) before harvesting and fixation as above. Fixed cells were washed in PBS-T and then resuspended in 200 µl Click reaction buffer (2 mM CuSO4, 50 mM L-ascorbic acid, 0.2 µl/ml Alexa Fluor^®^ 488 azide (Invitrogen Cat. No. A10266; 0.2 µg/µl solution) in PBS-T modified from (71) for 30 min at RT, washed in PBS-T, stained with DAPI as above and then analysed on a Cytoflex S analyzer with 450 nm laser and 525/50 filter for EdU detection.

### DNA combing

Exponentially growing MEFs and mESCs were pulse labelled by addition of 25 µM CldU (Sigma, Cat. No. C6891) for 20 min, washed with pre-warmed PBS and then pulsed with 125 µM IdU (Sigma, Cat. No. I7125) for 20 min. After trypsinization, 6×10^5^ cells were used to cast 3 agarose (Biorad, Cat. No. 1613111) plugs per condition and processed for DNA combing according to a previously described protocol (72, 73), omitting SCE buffer plug digestion steps. IdU and CldU were detected using mouse anti-BrdU (BD, Cat. No. 347580; RRID:AB_400326) and rat anti-BrdU (Abcam, Cat. No. ab6326; RRID:AB_305426), respectively. DNA was detected using anti-ssDNA antibody (Millipore, MAB 3034; RRID:AB_94645). Images were acquired on a widefield microscope (Zeiss Axiophot) with a 63X or 40X lens. The 2.33 Kbp/µm elongation rate (µm to Kbp conversion) was obtained from bacteriophage lambda DNA combing and measurement. Measurements and analysis were performed using ImageJ. DNA fork speed was obtained by dividing the length of the IdU tracks adjacent to CldU tracks (ongoing forks) by the IdU incubation time (20 min) and expressed in Kb/min. Fork asymmetry is presented as left IdU versus right IdU ratios. Interorigin distances (IODs) correspond to the space (in Kb) between the centre points of adjacent bi-directional replication origins.

Fork elongation experiment (fig. S16). Asynchronous DONSON-AID2 HCT116 cells were pulsed with 25 µM CldU for 20 min, washed with warm media, and incubated in new media containing 125 µM IdU with either vehicle or 15 µM 5-Ph-IAA for 25 min. Cells were subsequently trypsinized and processed for DNA combing as described above.

### Growth curves

MEFs (1.5 × 10^5^ cells) were seeded on day 0 into a T25 flask, split and counted every 3 days, and 1.5 × 10^5^ cells were reseeded into a new flask. mESCs (5 × 10^5^ cells) were seeded on day 0 into a T25 flask and split, counted and reseeded every 48 h at the same density. MEFs and mESCs were grown in 3% O_2_. Counts were measured in duplicate using a Countess automated cell counter according to manufacturer’s instructions. Doubling times were calculated during log-phase growth (day 3 to 36 for MEFs and day 2 to 16 for mESCs) using the formula: *t*/log2 (*e*/b) where t = time in hours, e = final population size and b = population size at the start of log-phase growth.

### Generation of *Donson*^*M440T/M440T*^ knock-in mice using CRISPR

Mouse studies were approved by the University of Edinburgh animal welfare and ethical review board and conducted according to UK Home Office regulations under UK Home Office project licenses P2A477A62 and PP2060675. Fertilized eggs were injected with a CRISPR mix targeting *mDonson* containing 50 ng/μl Cas9 mRNA (Trilink, Cat. No. L-6125), 25 ng/μl gRNA (TACCTTAAGCATTTGCATTG) and 150 ng/μl single-stranded oligodeoxynucleotide repair template (ssODN, IDT ultramer, 5’-AAATTGCTGGACTGTATGTAGATGAAGTAAACACTTACCTTAAGCATTTGCGTTGATGCACCTCGGAAGGCTATTGGGGATAAGAGAGTGGGTGGGAGACCTGCCTGT-3’) in nuclease-free water. Injected eggs cultured overnight up to the two-cell stage were transferred to the oviducts of pseudo-pregnant females. Although the repair template contained a silent PAM site mutation (c.1314C>A) besides the desired c.1319T>C substitution (*p.M440T*), a founder mouse without the PAM site mutation was selected to establish the mouse line which was maintained on a mixed CD1/BL6CBAF1 background. WT and Donson^M440T/M440T^ MEFs (Mus musculus, mixed background CD1/BL6CBAF1) were derived from embryos and immortalized using CRISPR to delete p53.

### Embryo measurements

Dissected embryos were washed in ice-cold PBS for 5 min and fixed with ice-cold 4% Paraformaldehyde/PBS overnight at 4°C, washed several times in PBS, and imaged using a stereomicroscope. In lateral images, occipital-frontal distance was measured from nasion to occiput and embryo length was measured from bregma to the most caudal point of the embryo (excluding hindlimbs and tail); scale bars indicated in figures and legends. For cortical thickness analysis, embryos were cryopreserved in 30% Sucrose/PBS, embedded in Tissue-Tek OCT (Sakura Cat. No. 4583) and 12 µM coronal cryostat sections were obtained and stored at −70°C. After PBS washes, sections containing brain tissue were stained for 10 min in 0.1 µg/ml DAPI solution in PBS for 5 min, washed in PBS and mounted using Mowiol 4–88 (Sigma Cat. No. 81381). Embryos/samples allocated to groups on basis of genotypes. No statistical method was used to predetermine sample size. Sample size was chosen based on standard practices in the field, no blinding of measurements were done, no data was excluded.

### Alcian-blue Alizarin-red stainings

Embryos were fixed in 100% ethanol for 2 days, placed in acetone for 3 days, and stained O/N at 37°C in staining solution containing 1 ml 0.3% alcian blue (Sigma Ca. No. 05500) in 70% ethanol, 1 ml 0.1% alizarin red (Sigma Ca. No. 5533) in 95% ethanol, 1 ml glacial acetic acid and 17 ml 70% ethanol. After 2 washes in ethanol, embryos were cleared in 1% KOH (w/v), 1% KOH/10% glycerol and finally stored in 10% glycerol/PBS until imaging using a stereoscope (Leica).

### Statistical methods used in mammalian cell experiments

Statistical testing was performed using GraphPad Prism v.9.1.1. Two-sided parametric (t-tests) or non-parametric Mann-Whitney *U*-tests were performed for quantitative measurements as indicated in figure legends. Two-way ANOVA tests were performed for S-phase-entry assays; significance (*P* values) indicated on figures. Number of samples and/or experimental replicates indicated on figures or legends. No exclusion criteria were pre-established. No data/sample points were omitted.

## Supplementary Material

Data S1

Data S2

Table S2

Table S1

Table S3

1
