# Supplementary material for: In silico protein interaction screening uncovers DONSON’s role in replication initiation
Source: Science. Author manuscript; Available in PMC 2024 Jan 22. (PMC10801813; doi:10.1126/science.adi3448)
Supplement: 1 [file NIHMS1946428-supplement-1.pdf]

Supplementary Materials for

In silico protein interaction screening uncovers DONSON's role in vertebrate CMG helicase assembly

Authors: Yang Lim[1]†, Lukas Tamayo-Orrego[2]†, Ernst Schmid[1], Zygimante Tarnauskaite[2]‡, Olga V. Kochenova[1,3], Rhian Gruar[1], Sachiko Muramatsu[4], Luke Lynch[5], Aitana Verdu Schlie[2], Paula L. Carroll[2], Gheorghe Chistol[6], Martin A.M. Reijns[2], Masato T. Kanemaki[4,7,8], Andrew P. Jackson[2]*, Johannes C. Walter[1,3]*

Corresponding authors: johannes_walter@hms.harvard.edu, andrew.jackson@ed.ac.uk

The PDF file includes:

Figs. S1 to S21

Captions for Data S1 to S2

Other Supplementary Materials for this manuscript include the following:

Tables S1 to S3

Data S1 to S2

MDAR Reproducibility Checklist

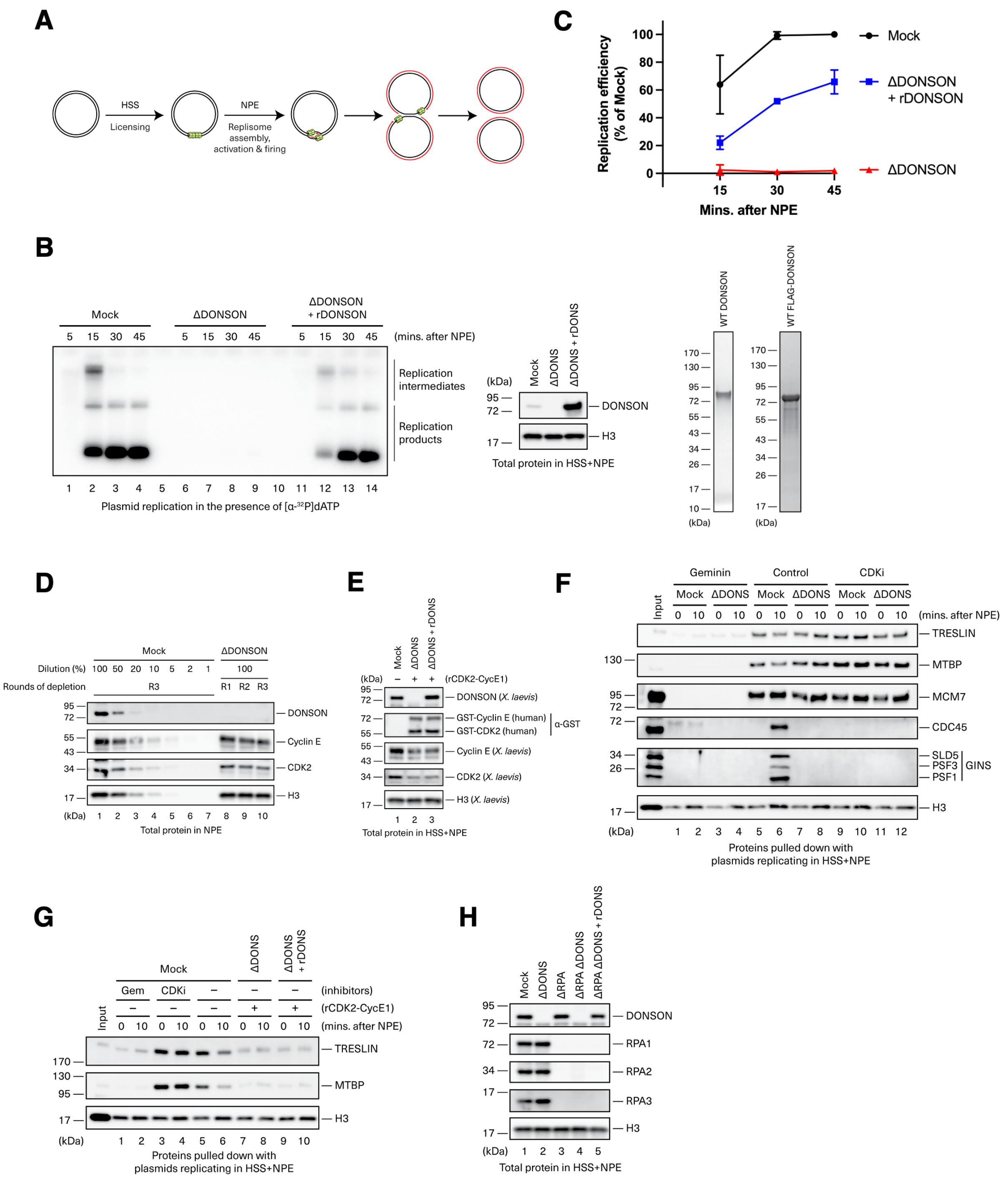

Fig. S1. DONSON depletion inhibits CMG assembly and partially co-depletes CDK2-Cyclin E. (A) Scheme depicting plasmid replication in frog egg extracts. HSS, high speed supernatant that supports replication licensing. NPE, nucleoplasmic extract that supports replication initiation from licensed DNA. **(B)** Left Panel: Plasmid DNA was replicated in the presence of radioactive [α-^{32}P]dATP as depicted in (A) using extracts that were either mock-depleted, DONSON-depleted, or DONSON-depleted with recombinant DONSON (rDONSON) added back to NPE. Replication products were separated on a native agarose gel and analyzed by autoradiography. Middle Panel: Total protein samples from the replication reactions in the left panel were blotted for the indicated

proteins. Right Panel: Purified recombinant DONSON and FLAG-DONSON were resolved by SDS-PAGE and visualized by Coomassie staining. Untagged DONSON was used for the rescue experiments in (B) and (C), and FLAG-DONSON was used for all other rescues and immunoprecipitation experiments. **(C)** The radioactivity in panel (B) and in two additional repeats was quantified and graphed. In the absence of added CDK2-Cyclin E, which was co-depleted with DONSON (see panels D and E), recombinant DONSON incompletely rescued DNA replication in DONSON-depleted extract. Datapoints, n=3 experiments. Mean±SD. **(D)** A dilution series of mock-depleted extract and DONSON-depleted extract after each round (R1-R3) of depletion were blotted for the indicated proteins. DONSON depletion removes ~50% of the endogenous CDK2-Cyclin E. **(E)** Mock-depleted extract (which contains 0.5-1 μM endogenous CDK2-Cyclin E) and DONSON-depleted egg extracts (where ~50% of the CDK2-Cyclin E was depleted) were optionally supplemented with 0.3 μM recombinant human CDK2-Cyclin E1 and blotted as indicated, including with anti-GST antibody to detect the recombinant CDK2 and Cyclin E1, and with anti-*Xenopus* Cyclin E and CDK2 antibodies to detect endogenous Cyclin E and CDK2, respectively. As in (D), DONSON depletion removes ~50% of the endogenous CDK2-Cyclin E. The protein levels shown here are representative of all the repeats presented in Fig. 1C-D. **(F)** Mock-depleted or DONSON-depleted egg extracts were supplemented with Geminin to inhibit licensing or $p27^{Kip}$ (CDKi) to block CDK2 activity (and no rCDK2-Cyclin E1 was added to DONSON-depleted extract). At the specified times following NPE addition, chromatin was recovered and blotted for the indicated proteins. DONSON depletion abolished CMG assembly (as seen in Fig. 1D, where the depleted extract was supplemented with rCDK2-Cyclin E1). Importantly, in the absence of added rCDK2-Cyclin E1, chromatin-bound TRESLIN and MTBP levels were the same in mocked-depleted and DONSON-depleted egg extracts (compare lanes 5-6 and 7-8), and went up slightly in the presence of CDKi (lanes 9-12). Thus, it appears that CDK2 de-stabilizes TRESLIN and MTBP on chromatin. **(G)** Samples from the experiment shown in Fig. 1D were blotted for TRESLIN and MTBP. The CDK-dependent destabilization of TRESLIN and MTBP binding shown in (F) probably explains why here, compared to mock-depleted extract (lanes 5 and 6), TRESLIN and MTBP chromatin levels are lower in DONSON depleted extract supplemented with rCDK2-Cyclin E1 (lanes 7 and 8). Importantly, in this condition, there is no difference in TRESLIN-MTBP levels in the presence and absence of DONSON (lanes 7-10). The H3 panel is a duplicate of that shown in Fig. 1D. **(H)** Western blot of total protein levels in reactions assayed by plasmid pull-down in Fig. 1E.

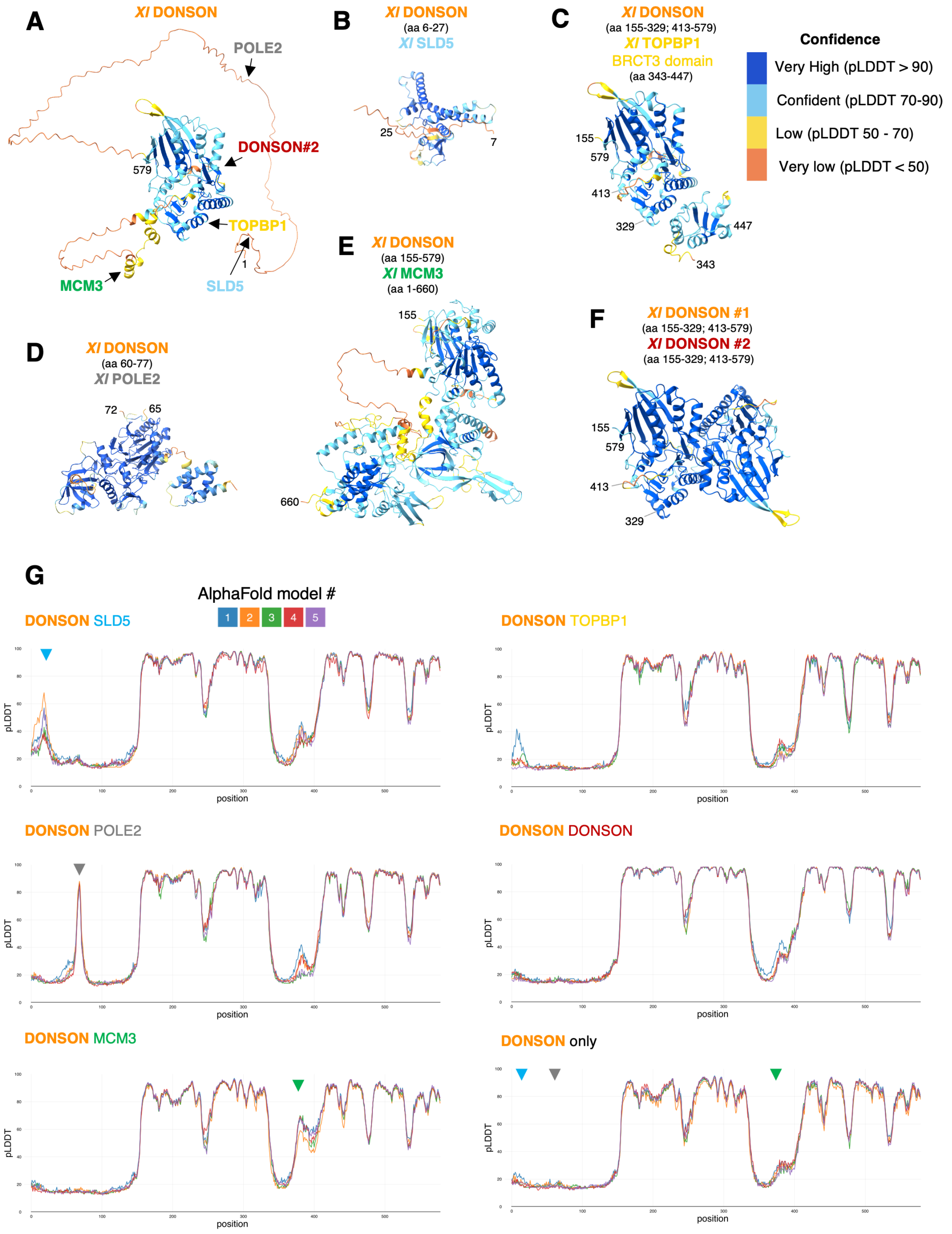

Fig. S2. Structures of the predicted Xenopus DONSON protein-protein interactions colored by pLDDT values. (A-F) The same structures shown in Fig. 2A-F are colored by pLDDT value, a measure of the confidence that an amino acid is positioned correctly relative to neighbouring residues. **(G)** pLDDT values for DONSON alone or in complex with each of its predicted partners

displayed as graphs. pLDDT values for each AF-M model are shown as differently colored lines. SLD5, POLE2, and MCM3, which are predicted to bind disordered regions of DONSON, induce large increases in pLDDT values at the predicted binding interface, which is evident from comparison with the corresponding pLDDT values of DONSON alone (colored arrowheads). TOPBP1 and DONSON generally have a lesser effect on DONSON pLDDT values because they are predicted to interact with regions of DONSON that are already well-ordered.

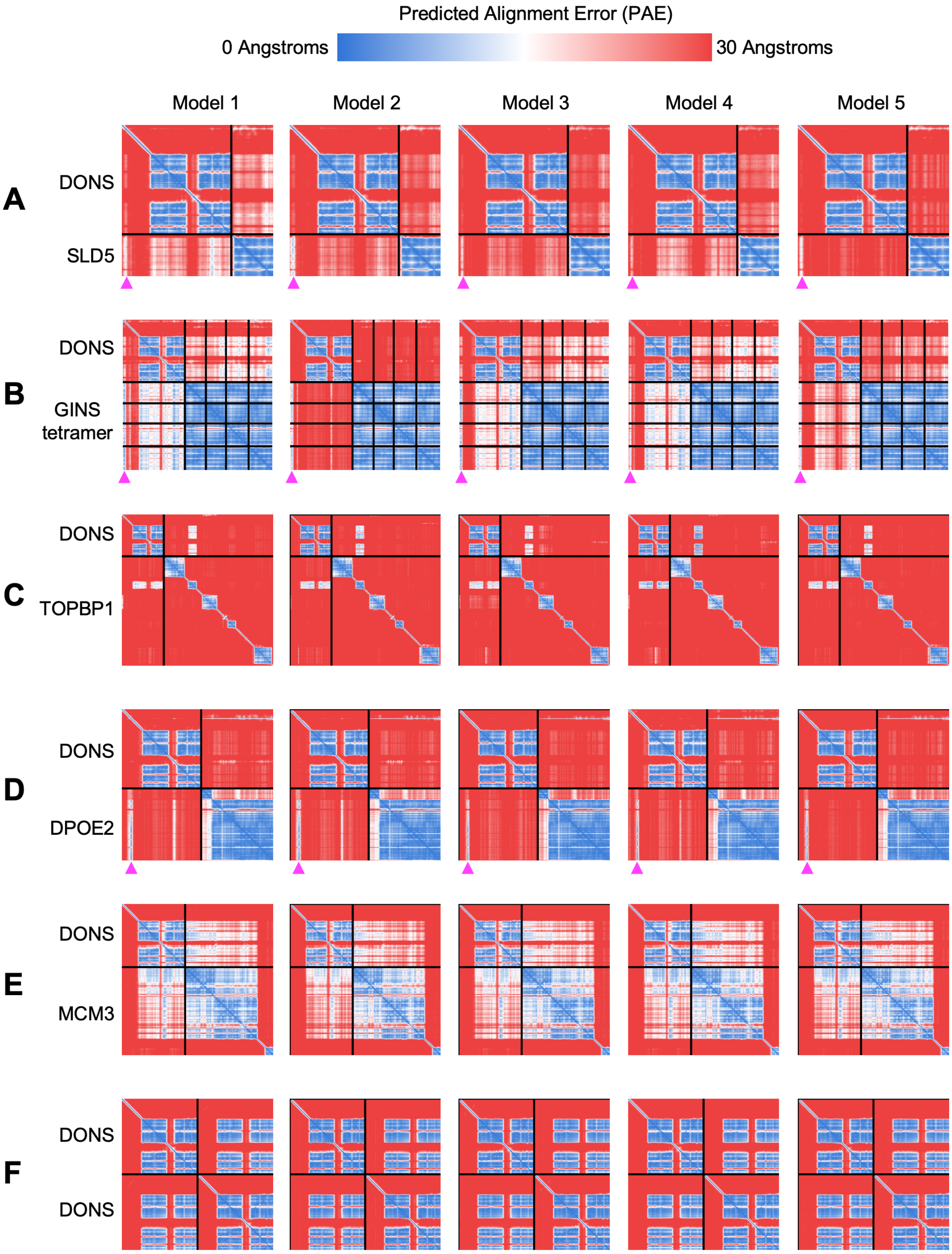

Fig. S3. Predicted alignment error (PAE) plots for DONSON and its predicted partners. PAE plots generated by the 5 AF-M models for the complex of DONSON with **(A)** SLD5, **(B)** the tetrameric GINS complex, **(C)** TOPBP1, **(D)** POLE2, **(E)** MCM3, and **(F)** DONSON itself. The peptide regions in the N-terminus of DONSON that are predicted to interact with SLD5, GINS,

and POLE2 are indicated with pink arrowheads. All proteins are from *Xenopus laevis*, and the PAE plots for human proteins are very similar.

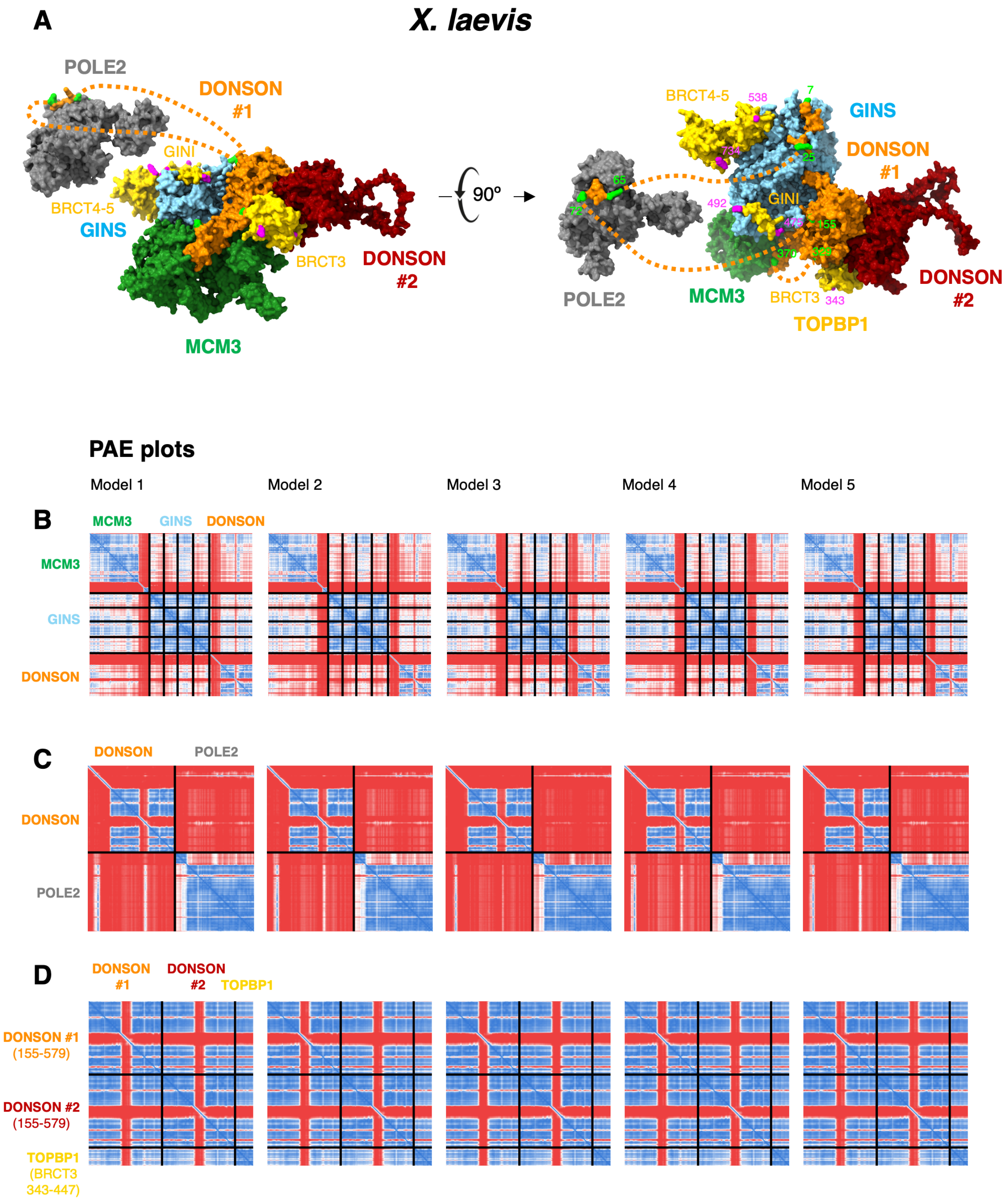

Fig. S4. ***Xenopus*** **DONSON is predicted to be able to bind all its partners simultaneously. (A)** AF-M was used to predict the structures of *Xenopus* MCM3-GINS-DONSON, TOPBP1-GINS, DONSON-DONSON-TOPBP1, and DONSON-POLE2. The first three structures were aligned to generate the complex shown with single copies of GINS, TOPBP1, MCM3, and two copies of DONSON (see methods for details). The DONSON-POLE2 complex is shown separately, but at the same magnification as the other complexes. This analysis revealed that DONSON's putative

interacting proteins are predicted to be able to all co-occupy DONSON. For DONSON#1 (orange), only the globular domain, the GINS-binding peptide, and the POLE2-binding peptide are shown. Dotted lines denote the flexible linkers attaching the POLE2 and GINS binding peptides to the globular domain. In DONSON #2 (brick red), only the globular domain is shown. For TOPBP1, the BRCT3, GINI, and BRCT4-5 regions are shown. Residues located at the ends of well-ordered DONSON and TOPBP1 segments are numbered and shown in green and pink, respectively. The complex is shown in two orientations. **(B-D)** The predicted alignment error plots are shown for complexes of MCM3-GINS-DONSON (B), DONSON-POLE2 (C), and DONSON(residues 155-579)-DONSON(residues 155-579)-TOPBP1 (residues 343-447; BRCT3 domain) (D).

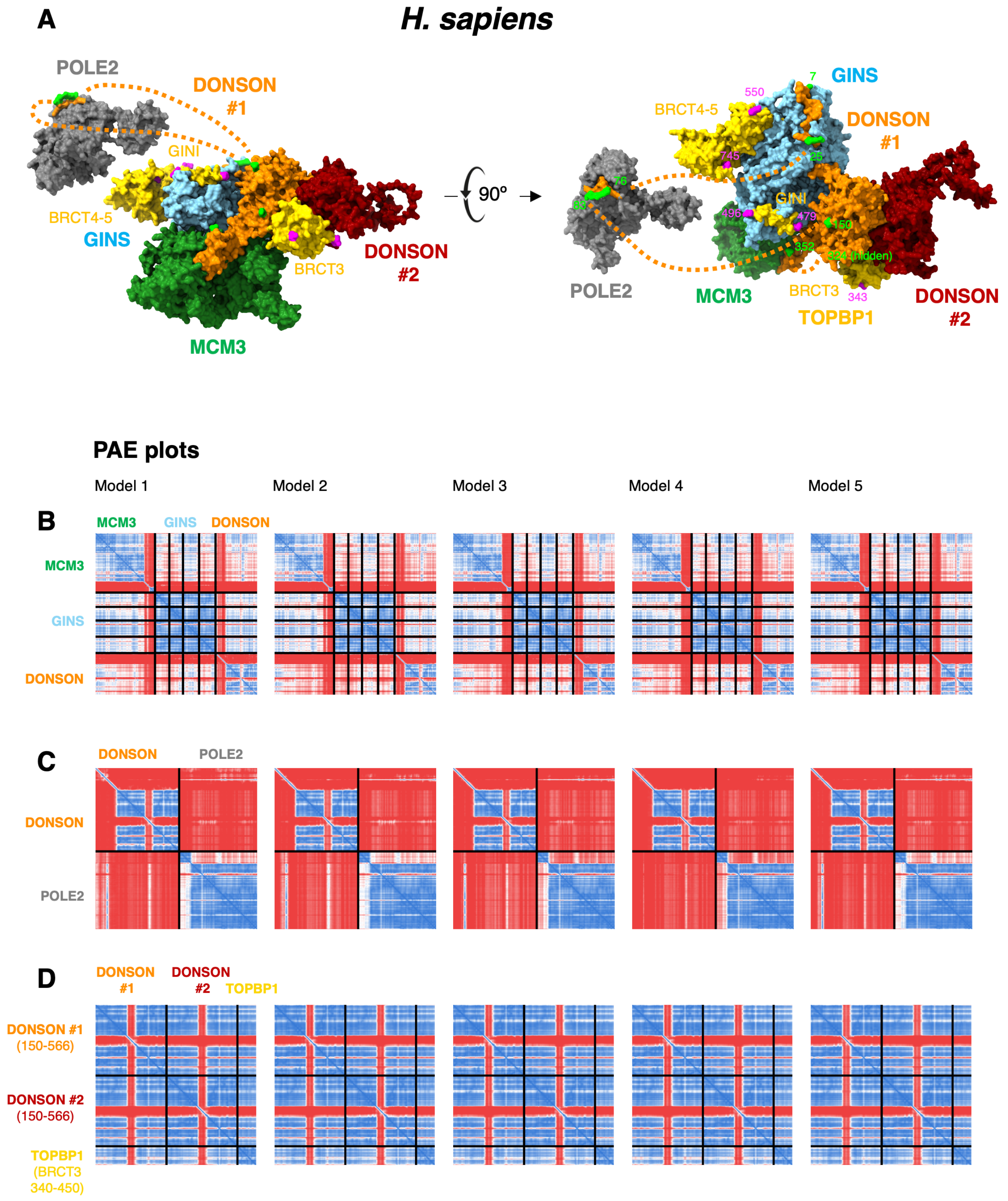

Fig. S5: Human DONSON is predicted to be able to bind all its partners simultaneously. (A-D) Same analysis as in fig. S5, but for human DONSON and interacting partners, demonstrating that the interaction of DONSON with its partners is predicted to be highly conserved in vertebrates.

5

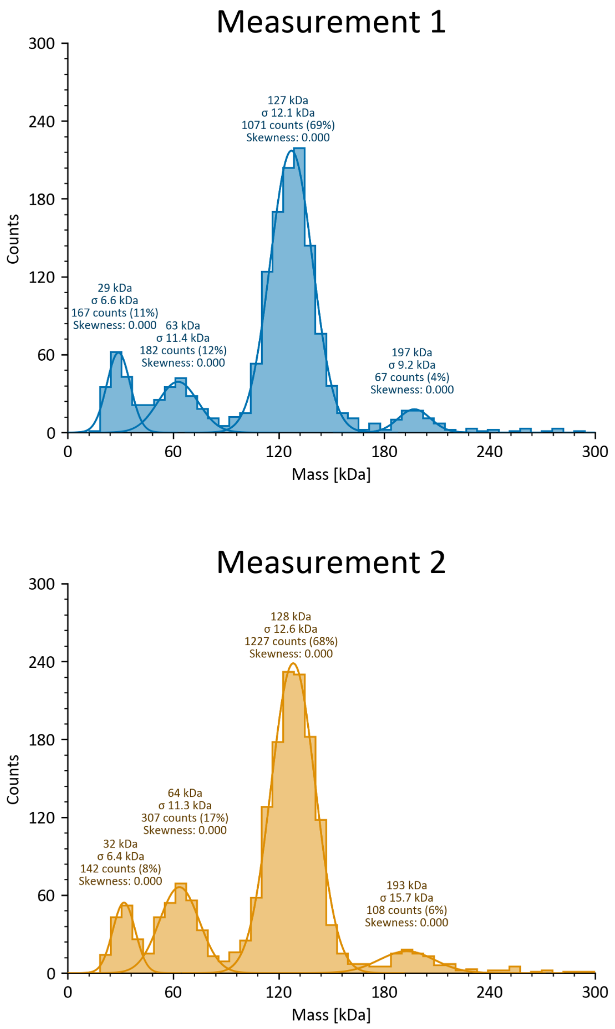

Fig. S6. Mass Photometry analysis of DONSON. Purified FLAG-DONSON was subjected to mass photometry analysis. Two independent measurements show that the majority of DONSON has a mass of 127-128 kD, which is double its calculated molecular weight of 64 kD. The 32 kD peak probably corresponds to remnant glutathione S-transferase from the purification process.

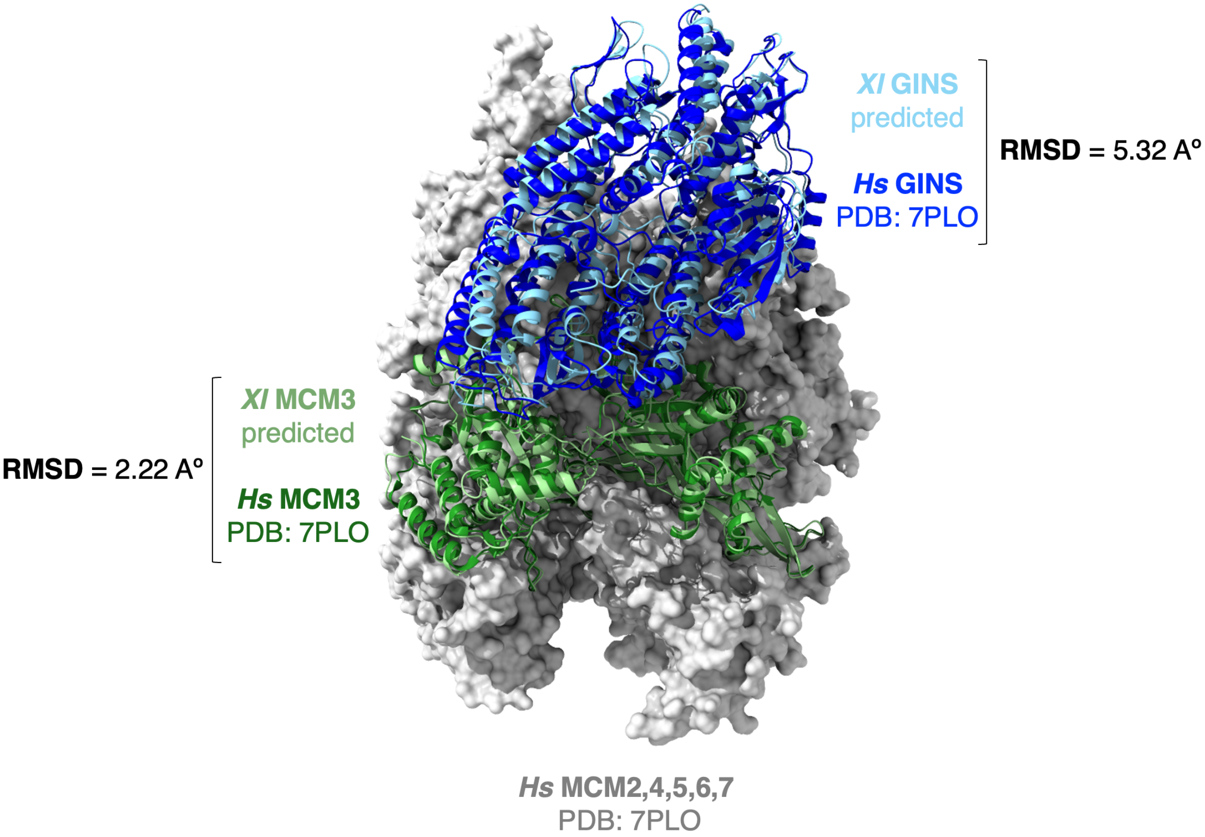

Fig. S7. DONSON is predicted to position GINS on MCM2-7 for CMG assembly. AF-M was used to predict a human MCM3-GINS-DONSON complex. This complex was superposed onto the cryo-EM structure of human CMG (PDB: 7PLO (*66*)) by aligning on the common MCM3 subunit. Only GINS and MCM3 of the predicted structure are shown, and all but GINS and MCM2-7 from 7PLO were deleted. ChimeraX was used to calculate RMSDs for GINS and MCM3, which are indicated. This analysis shows that DONSON docking of GINS onto MCM2-7 via MCM3 is predicted to place GINS very close to where it resides in CMG.

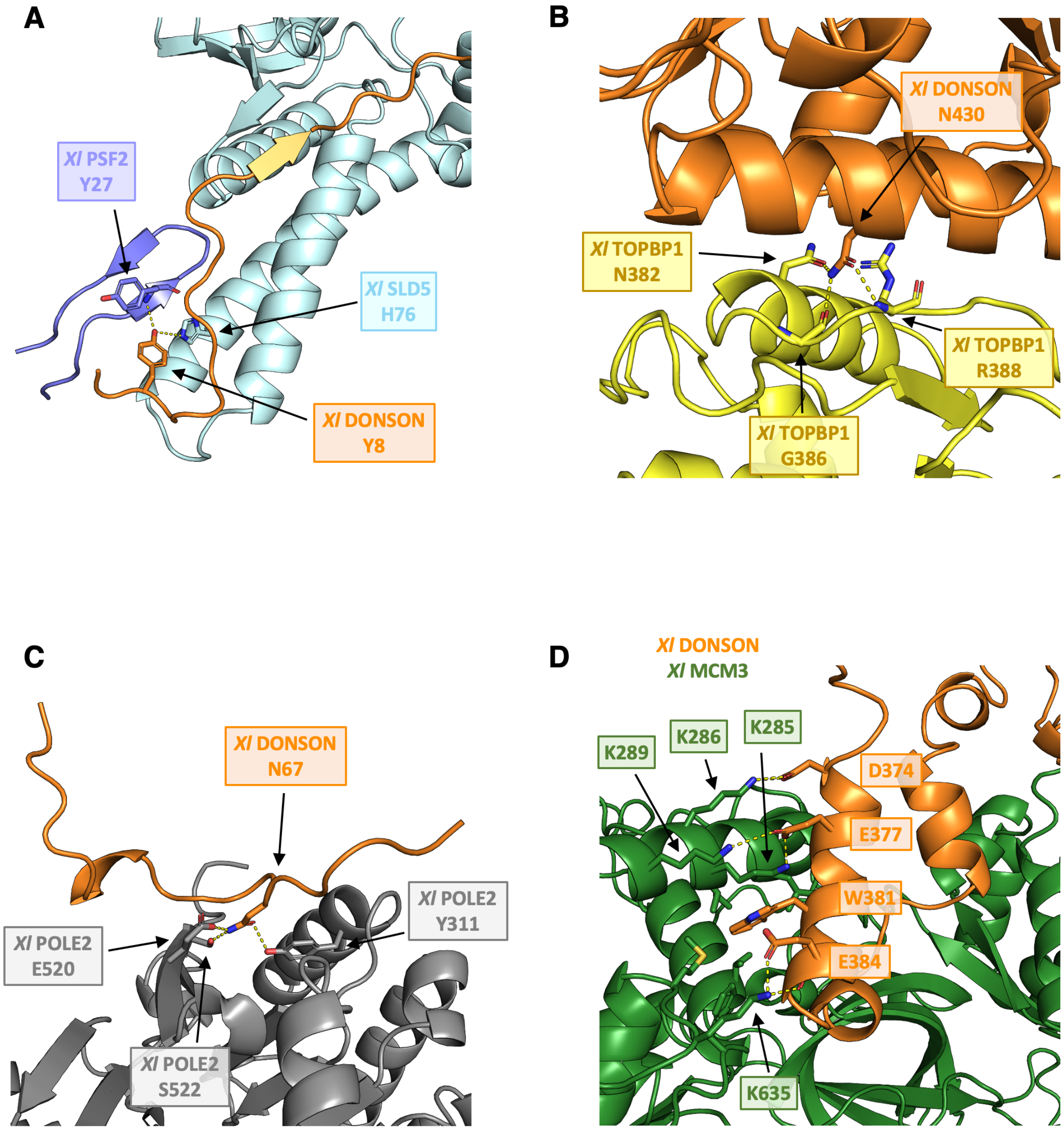

Fig. S8. Close-up views of key DONSON residues that were predicted to interact with other pre-LC components. AF-M predictions of complexes between DONSON and **(A)** GINS (with parts of its SLD5 and PSF2 subunits shown), **(B)** TOPBP1, **(C)** POLE2, and **(D)** MCM3, with mutated residues in DONSON and interacting residues in each partner shown as sticks. See Fig. 2A for overview of where mutations are located within DONSON.

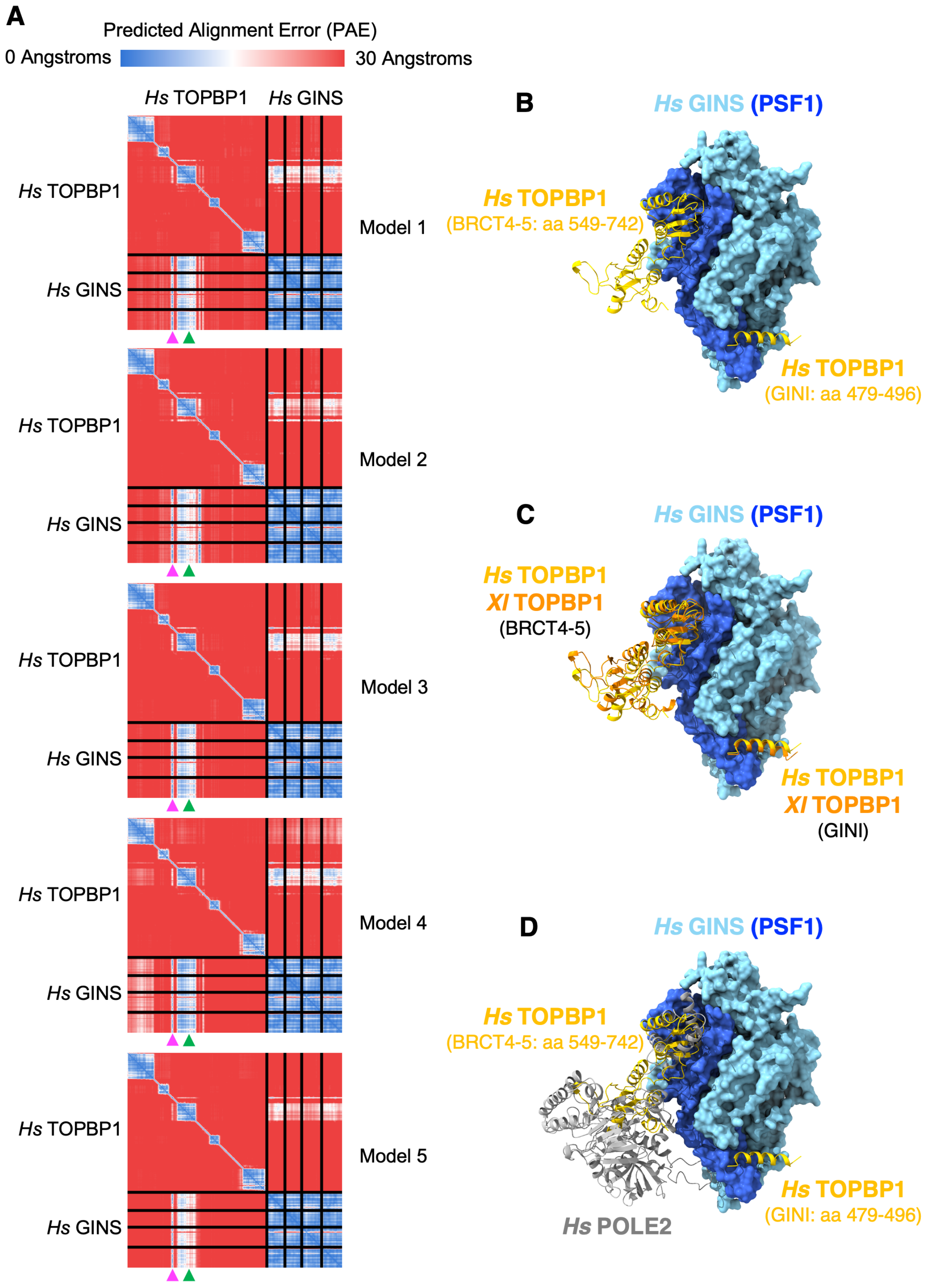

Fig. S9: TOPBP1 and POLE2 occupy the same site on GINS. (A) AF-M was used to predict structures of human TOPBP1 and GINS in all 5 AF-M models. The predicted alignment error (PAE) plots show that a peptide corresponding to the previously characterized GINI domain (pink arrowhead), as well as the BRCT4-5 domains (green arrowhead), are predicted with high confidence to interact with GINS in all 5 models (avg_n_models = 4.1; max_n_models = 5;

best_model_avg_plddt = 80.3; best_model_pdockq = 0.711; best_model_avg_pae = 6.8; see table S1 for explanation of metrics). **(B)** The rank 1 model from (A) is displayed (but all 5 models are very similar), with GINS shown in blue as a space filling model and TOPBP1 shown in gold as a ribbon diagram. The model reveals that BRCT4-5 and the GINI motif bind to different parts of PSF1 (dark blue). For clarity, only the domains in TOPBP1 that bind GINS are shown (GINI, residues 479-496; BRCT4-5, residues 549-742). We deleted residues 1-14 in SLD5 of the GINS complex, which are disordered. The structure agrees with the recently reported cryo-EM structure of the TOPBP1-GINS complex (*36*). **(C)** Similarity of Xenopus and human TOPBP1-GINS complexes. Xenopus TOPBP1 and GINS were folded using AF-M, which yielded a high confidence interaction (avg_n_models = 3.2; max_n_models = 5; best_model_avg_plddt = 81.5; best_model_pdockq = 0.712; best_model_avg_pae = 5.2). All TOPBP1 residues but 475-492 (GINI domain) and 538-734 (BRCT4-5 domain) were deleted from the top-ranked Xenopus TOPBP1-GINS model, and SLD5 residues 1-14 were deleted. The resulting structure was aligned on GINS from the human TOPBP1-GINS complex shown in (A), yielding excellent *Xl*GINS-*Hs*GINS overlap. Xenopus GINS was hidden, revealing that Xenopus and human TOPBP1 proteins interact similarly with GINS. **(D)** TOPBP1 and POLE2 binding to GINS is incompatible. The cryo-EM structure of the human replisome (7PLO) was aligned with the structure shown in (B) using GINS, and everything in the human replisome was deleted except POLE2 (grey). This shows that TOPBP1 and POLE2 bind to the same surface of GINS.

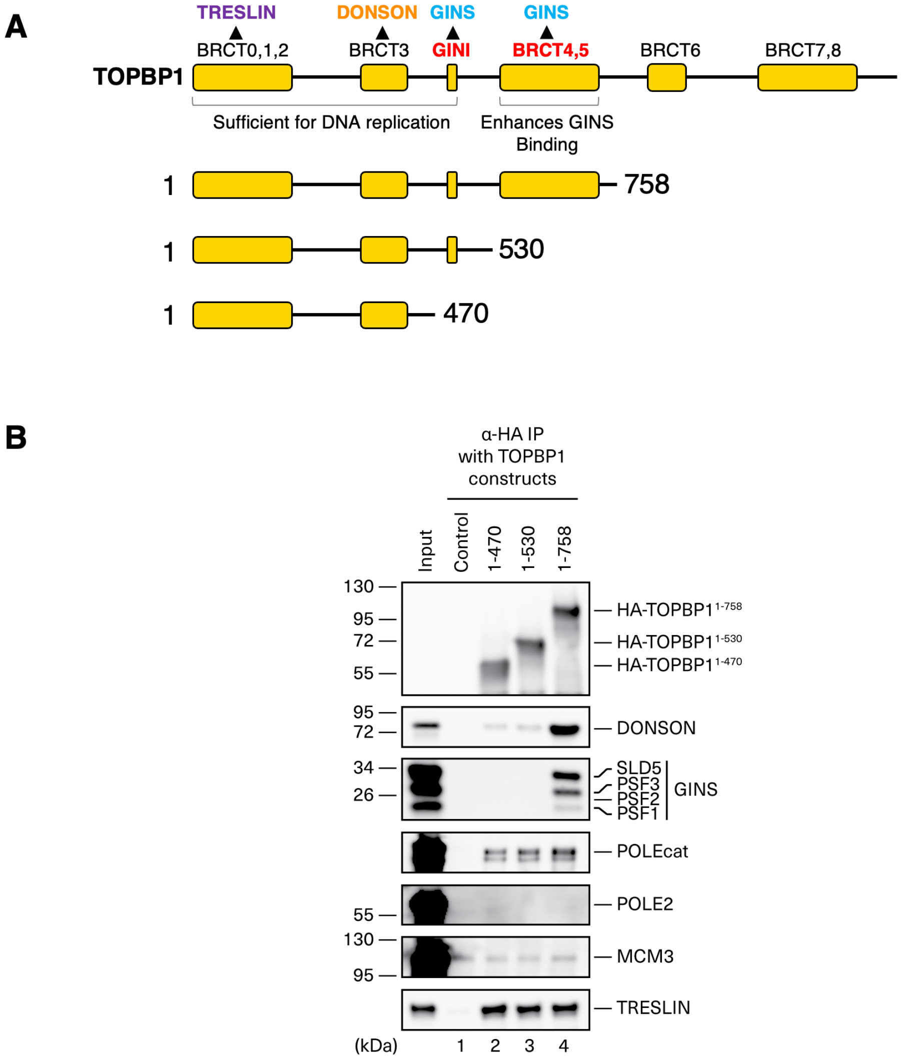

Fig. S10. TOPBP1's BRCT4-5 domain enhances TOPBP1 binding to GINS and DONSON. (A) Truncated constructs of TOPBP1 depicted in reference to the schematic showing the functional domains of TOPBP1 from Fig. 2G. **(B)** The indicated HA-TOPBP1 proteins were expressed in wheat germ extract, pre-immobilized on anti-HA antibody beads, and used for recovery of binding partners from non-replicating NPE. The presence of BRCT4-5 ($TOPBP1^{1\text{-}758}$) greatly enhanced the recovery of DONSON and GINS.

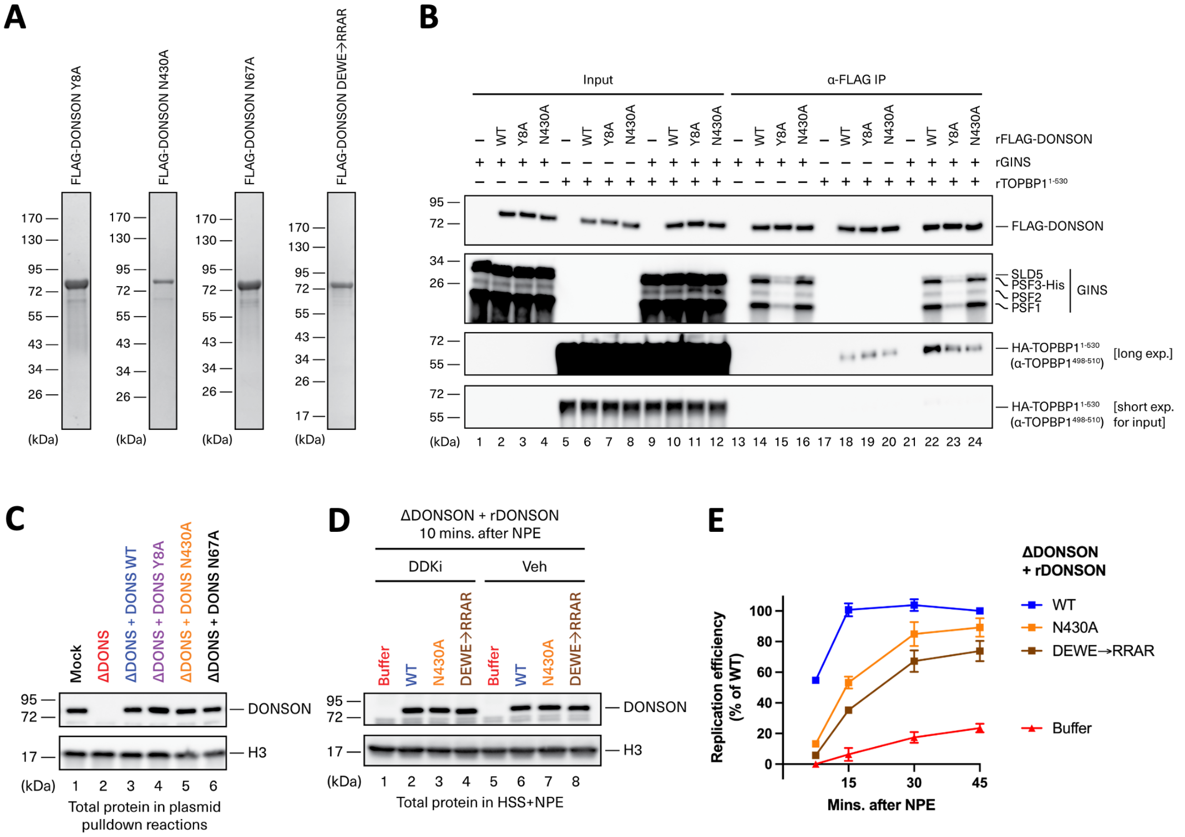

Fig. S11. Supplementary information about purified DONSON mutants and reconstitution of pre-LC formation. **(A)** Purified recombinant FLAG-DONSON containing the indicated mutations were resolved by SDS-PAGE and visualized by Coomassie staining. These DONSON proteins were used in Figs. 3 and 4, as well as panels (B)-(E) in this figure. **(B)** The indicated FLAG-DONSON proteins (fig. S1B and panel (A) in this figure) were optionally mixed with purified GINS or TOPBP1$^{1\text{-}530}$, as indicated. DONSON was recovered using FLAG IP and blotted for the indicated proteins alongside the input reactions. In the absence of GINS, DONSON recovered the same level of TOPBP1 as seen for DONSON^{Y8A} in the presence of GINS (lanes 18 and 23), consistent with efficient TOPBP1 binding to DONSON being dependent on GINS. **(C)** Western blot of total protein levels in reactions assayed by plasmid pull-down in Fig. 4B. The protein levels are also representative of all the repeats presented in Fig. 4A. **(D)** Western blot of total protein levels in reactions assayed by plasmid pull-down in Fig. 4D. The protein levels are also representative of all the repeats presented in panel (E) in this figure. **(E)** Egg extracts were depleted of DONSON, supplemented with rCDK2-Cyclin E1 and the indicated DONSON proteins, and used to measure DNA replication. Datapoints, n=3 experiments, except the 7.5 min timepoint where n=1. Mean±SD.

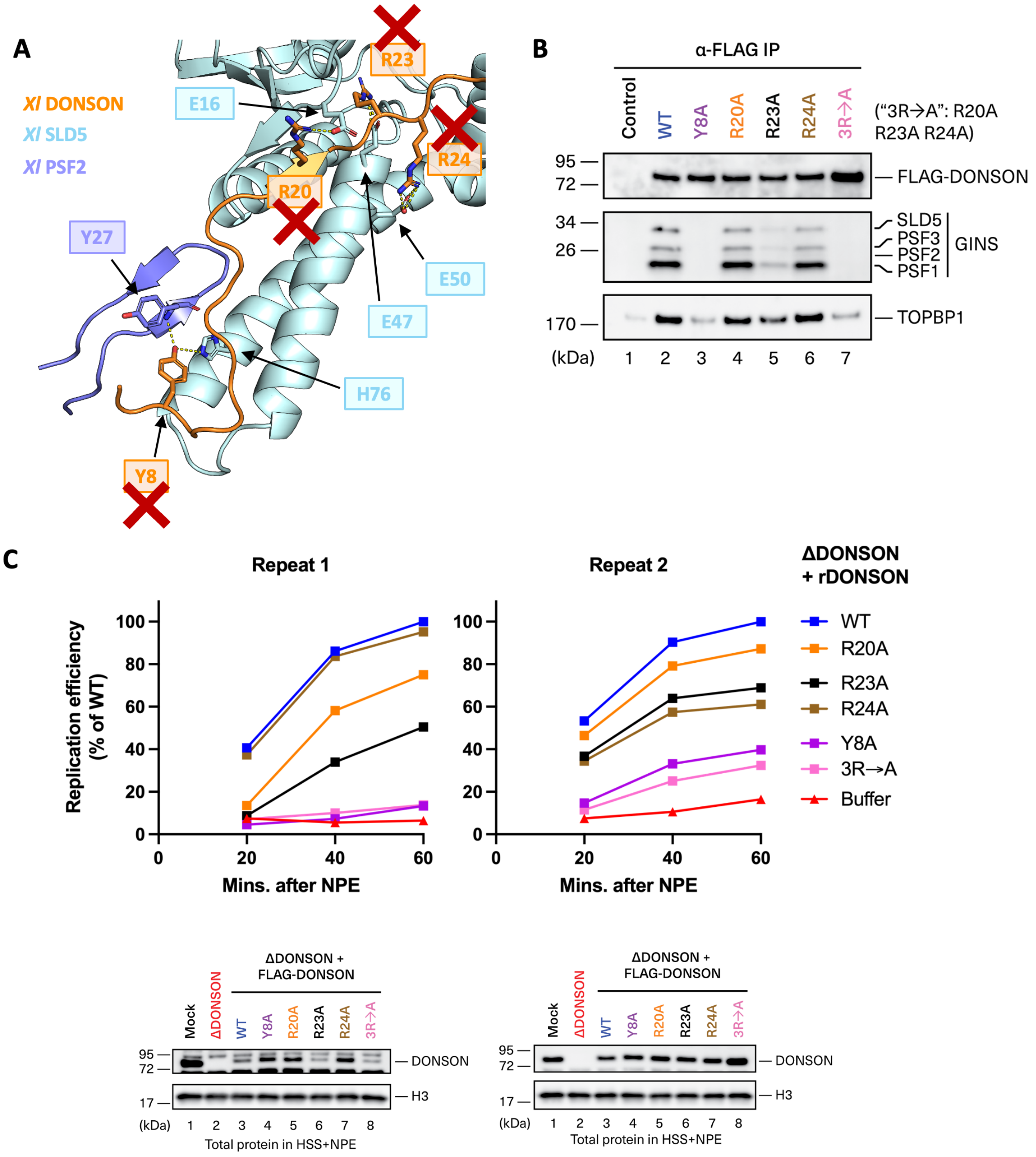

Fig. S12. Mutagenesis of additional DONSON residues at the DONSON-GINS interface disrupts DNA replication. (A) AF-M prediction of the complex between DONSON and GINS (parts of its SLD5 and PSF2 subunits shown), with mutated residues in DONSON and interacting residues in GINS shown as sticks. Red crosses indicate residues mutagenized in this figure. **(B)** The indicated FLAG-DONSON proteins were expressed in wheat germ extract, pre-immobilized on anti-FLAG beads, and used for recovery of binding partners from non-replicating NPE. The results show the effect of each DONSON mutant on pre-LC assembly. **(C)** Egg extracts were depleted of DONSON, supplemented with rCDK2-Cyclin E1 and the indicated DONSON proteins (expressed in wheat germ extract), and used to measure DNA replication. Two repeats of the experiment, as well as western blots of total protein levels in the replication reactions, are shown. The results show that DONSON^{R24A} and DONSON^{R20A} have the mildest effects on replication and

pre-LC assembly, the effect of R23A is stronger, and mutation of all three arginines (3R→A) has the strongest effect.

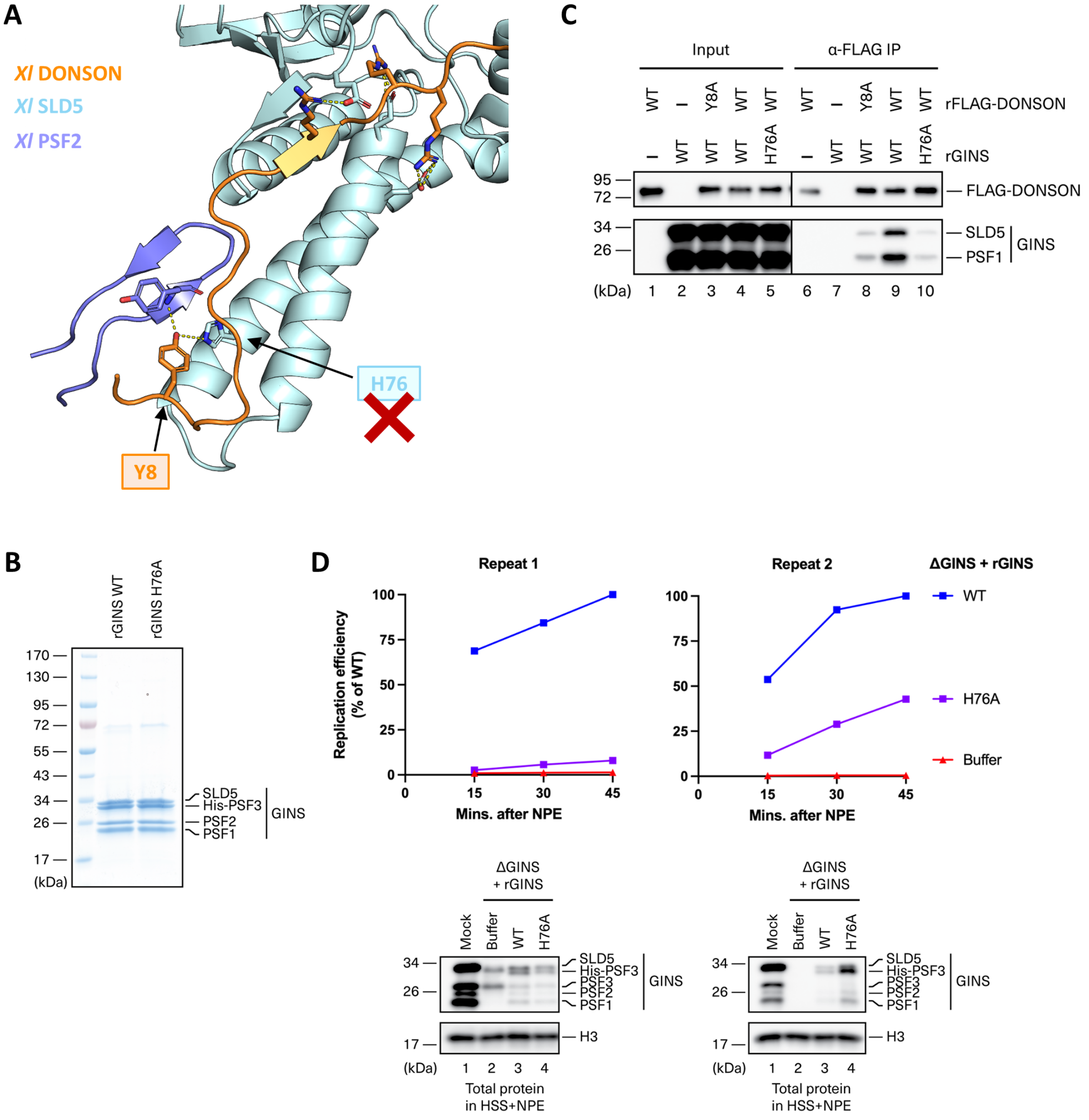

Fig. S13. Mutagenesis of SLD5 H76 at the DONSON-GINS interface disrupts DNA replication. (A) AF-M prediction of the complex between DONSON and GINS (parts of its SLD5 and PSF2 subunits shown), with interacting residues in DONSON and GINS shown as sticks. Red crosses indicate the residue mutagenized in this figure. **(B)** Coomassie stained SDS-PAGE gel showing purified recombinant $GINS^{WT}$ and $GINS^{SLD5\text{-}H76A}$. **(C)** The indicated purified FLAG-DONSON (fig. S1B) and GINS proteins (panel (B) in this figure) were mixed, and DONSON was recovered using FLAG IP. The eluates were blotted for the indicated proteins alongside the input reactions. The recovery of GINS was used as an indicator of the effect of each GINS or DONSON mutation on the DONSON-GINS interaction. $SLD5^{H76A}$ disrupts the DONSON-GINS interaction to the same extent as the reciprocal $DONSON^{Y8A}$ mutation. **(D)** Egg extracts were depleted of GINS, supplemented with the indicated purified recombinant GINS protein (from panel B), and used to measure DNA replication. Two repeats of the experiment, as well as western blots of total protein levels in the replication reactions, are shown. The results show that $GINS^{SLD5\text{-}H76A}$ has a

severe replication defect, even when supplemented at a higher concentration than $GINS^{WT}$ (repeat 2, right panel).

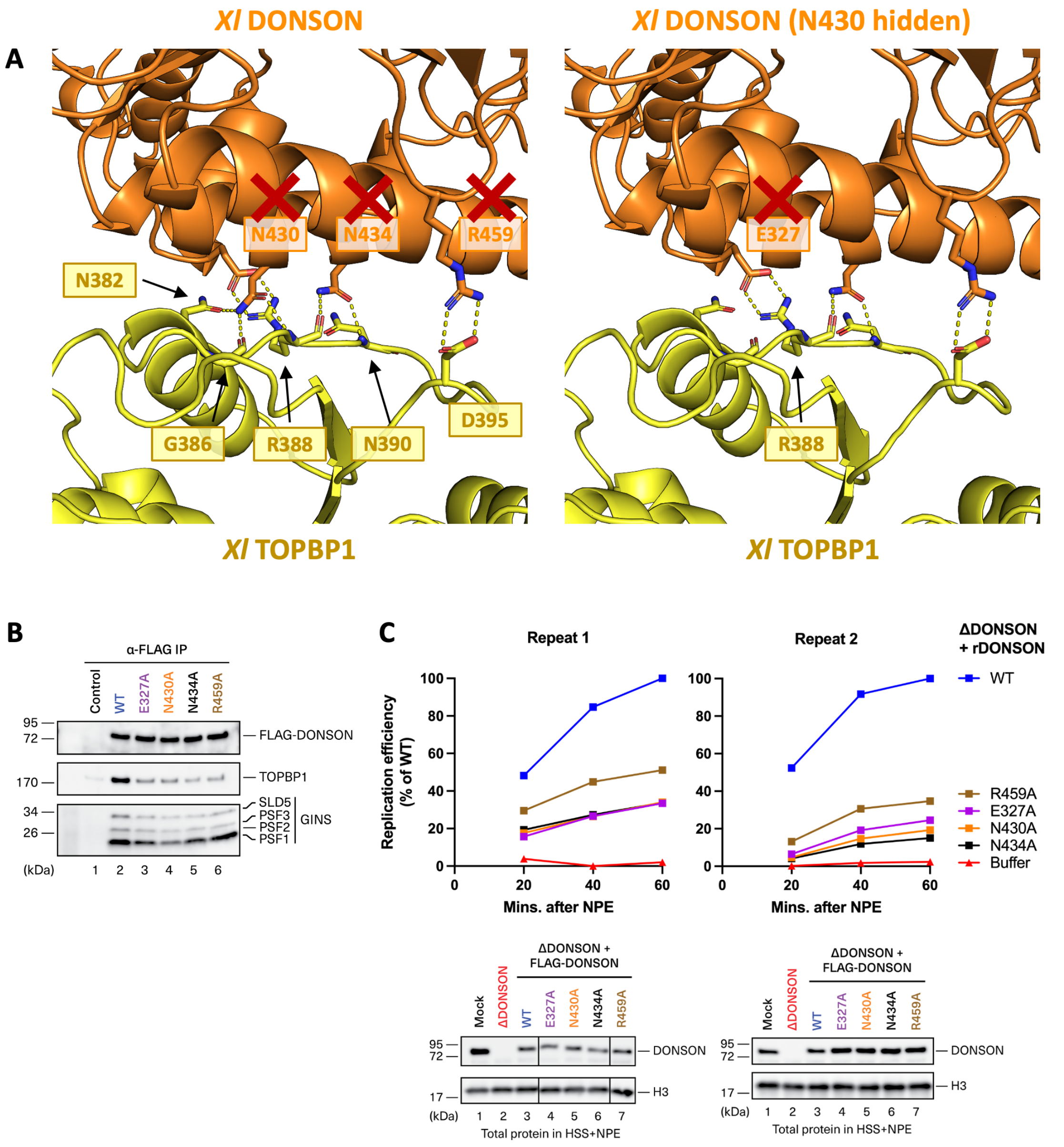

Fig. S14. Mutagenesis of additional DONSON residues at the DONSON-TOPBP1 interface disrupts DNA replication. (A) AF-M prediction of the complex between DONSON and TOPBP1, with mutated residues in DONSON and interacting residues in TOPBP1 shown as sticks. Red crosses indicate residues mutagenized in this figure. **(B)** The indicated FLAG-DONSON proteins were expressed in wheat germ extract, pre-immobilized on anti-FLAG beads, and used for recovery of binding partners from non-replicating NPE. The results show the effect of each DONSON mutant on pre-LC assembly. **(C)** Egg extracts were depleted of DONSON, supplemented with rCDK2-Cyclin E1 and the indicated DONSON proteins (expressed in wheat germ extract), and used to measure DNA replication. Two repeats of the experiment, as well as western blots of total protein levels in the replication reactions, are shown. The results show that all the DONSON mutants disrupted DONSON co-IP with TOPBP1 and DNA replication. In the left panel showing total protein levels in the replication reactions, the images are part of the same western blot, which was cropped to remove irrelevant information between lanes 3–4 and 6–7.

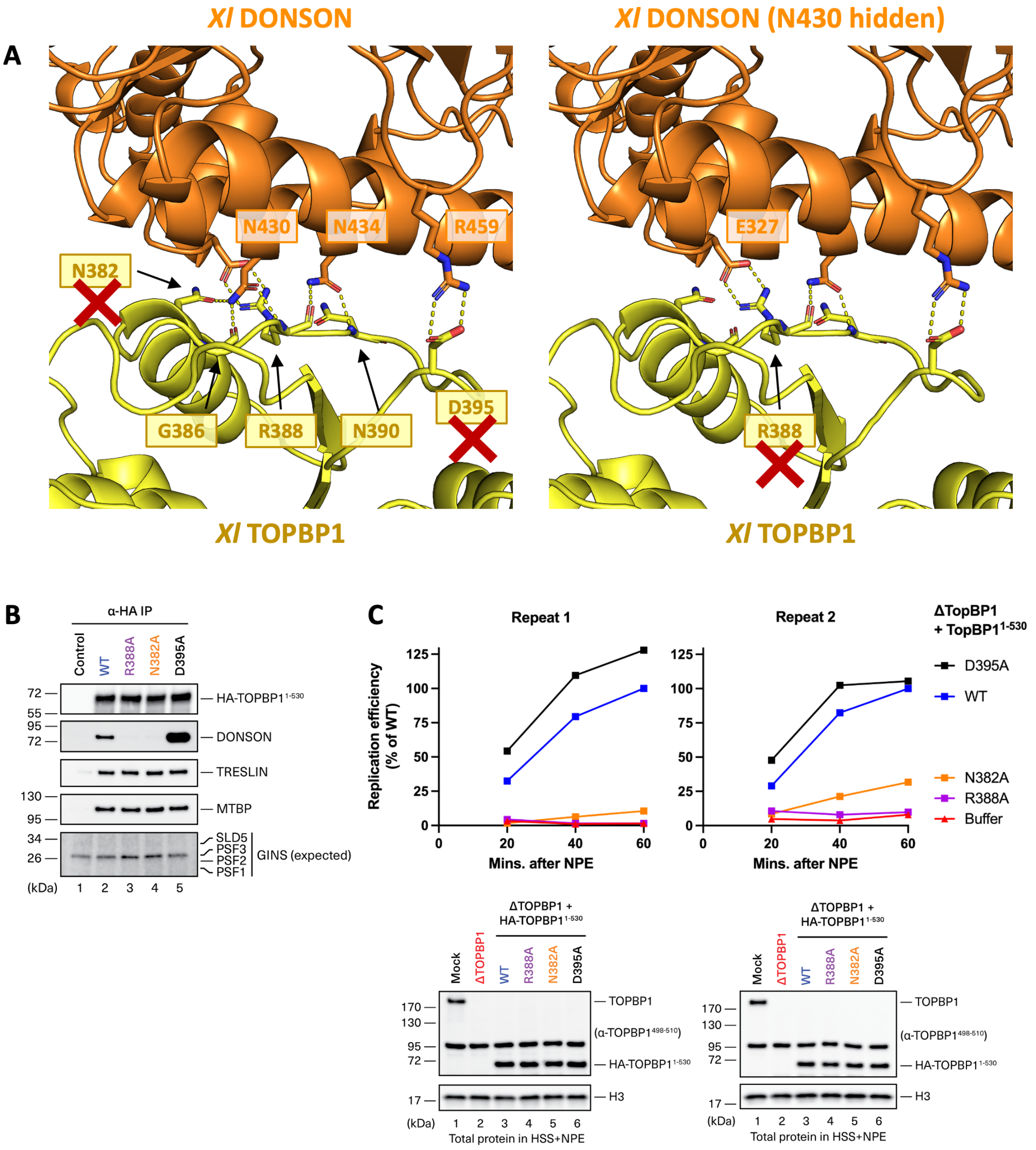

Fig. S15. Mutagenesis of TOPBP1 residues at the DONSON-TOPBP1 interface disrupts DNA replication. (A) AF-M prediction of the complex between DONSON and TOPBP1, with mutated residues in TOPBP1 and interacting residues in DONSON shown as sticks. Red crosses indicate residues mutagenized in this figure. **(B)** The indicated HA-TOPBP1$^{1-530}$ proteins were expressed in wheat germ extract, pre-immobilized on anti-HA antibody beads, and used for recovery of binding partners from non-replicating NPE. The results show the effect of each TOPBP1$^{1-530}$ mutant on pre-LC assembly. As shown in fig. S10, TOPBP1$^{1-530}$ inefficiently co-IPs GINS. **(C)** Egg extracts were depleted of TOPBP1, supplemented with the indicated TOPBP1$^{1-530}$ proteins (expressed in wheat germ extract), and used to measure DNA replication. Two repeats of the experiment, as well as western blots of total protein levels in the replication reactions, are shown. There was an excellent correlation between TOPBP1's ability to co-IP DONSON and to

support DNA replication. The correlation extends to $TOPBP1^{D395A}$, which appears to be a gain of function mutant.

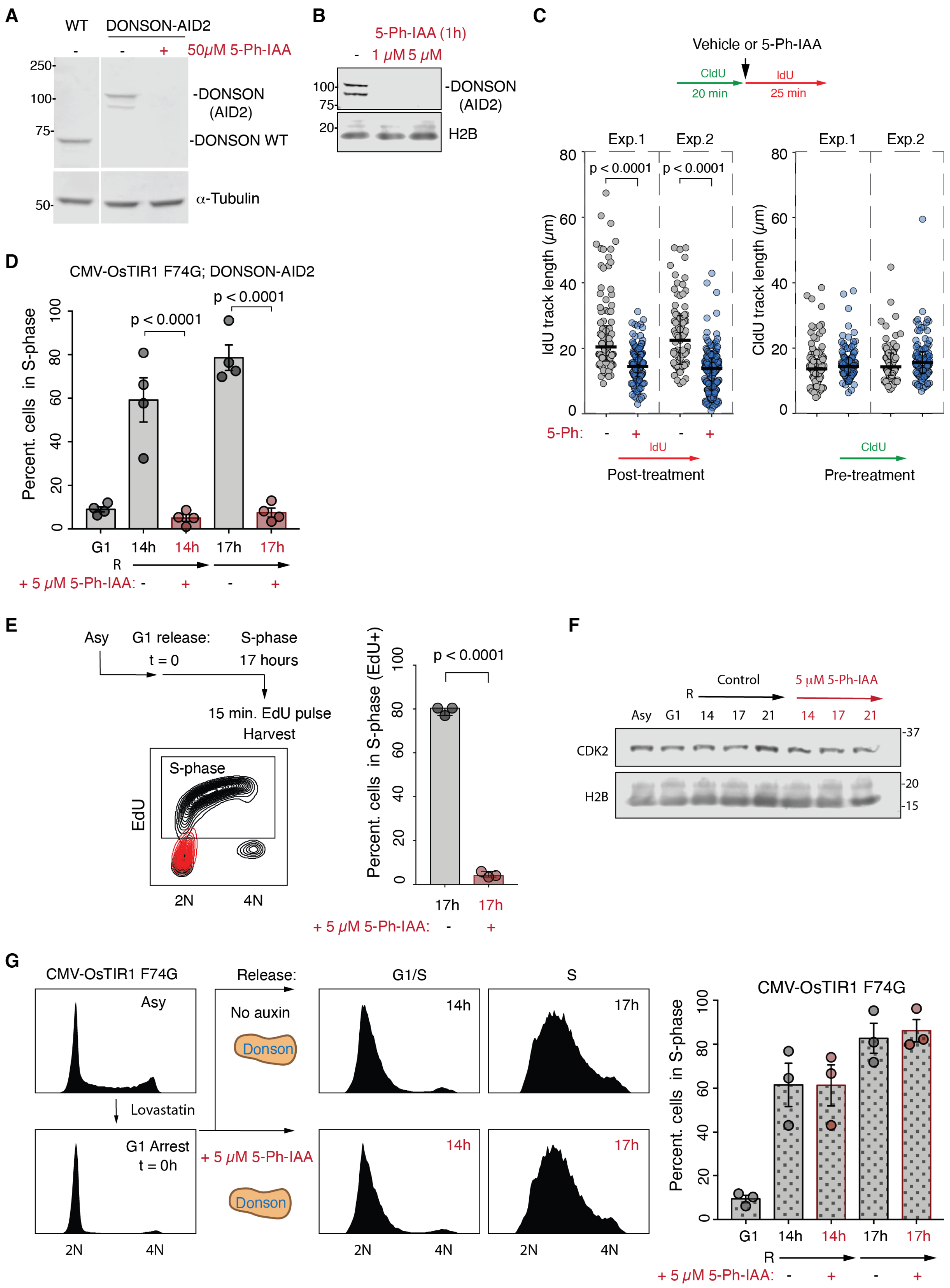
A
WT
DONSON-AID2
50μM 5-Ph-IAA
250-
100-
75-
50-
-DONSON (AID2)
-DONSON WT
α-Tubulin
B
5-Ph-IAA (1h)
1 μM 5 μM
-DONSON (AID2)
H2B
C
Vehicle or 5-Ph-IAA
CldU 20 min
IdU 25 min
Exp.1
Exp.2
p < 0.0001
IdU track length (μm)
CldU track length (μm)
5-Ph:
Post-treatment
Pre-treatment
D
CMV-OsTIR1 F74G; DONSON-AID2
Percent. cells in S-phase
G1
14h
17h
R
+ 5 μM 5-Ph-IAA:
E
Asy
G1 release: t = 0
S-phase 17 hours
15 min. EdU pulse
Harvest
S-phase
EdU
2N
4N
Percent. cells in S-phase (EdU+)
F
Control
5 μM 5-Ph-IAA
Asy G1 14 17 21 14 17 21
CDK2
H2B
G
CMV-OsTIR1 F74G
Asy
Lovastatin
G1 Arrest t = 0h
Release:
No auxin
Donson
+ 5 μM 5-Ph-IAA
G1/S
S
14h
17h

Fig. S16. 5 Ph-IAA treated DONSON-AID2 cells fail to initiate DNA replication. (A) Immunoblot of parental (WT) HCT116 CMV-OsTIR1 (F74G) cells and derived DONSON-AID2 cells in which endogenous DONSON was C-terminally tagged by CRISPR mediated genome editing to insert an AID2 tag (*38*). DONSON (62.7 kDa) in WT cells (lane 1) is not detected in the targeted line, replaced by DONSON-AID2 (97.7 kDa), confirming targeting of both alleles. DONSON antibody raised against N-terminal DONSON (AA1-125). DONSON is not detected after 50µM 5-Ph-IAA treatment for 24 hours in DONSON-AID2 cells and **(B)** effective depletion is evident at low doses for 1 hour duration. **(C)** DONSON is required for efficient fork elongation. Left, schematic of experiment, asynchronous DONSON-AID2 HCT116 cells pulse labelled with CldU, washed with media, then incubated in media containing IdU with or without 5-Ph-IAA. Right, quantification of DNA combing. Elongation rate of ongoing forks during DONSON depletion plotted as IdU track lengths. Individual data points, track lengths. Mean±SEM; n= 2 independent experiments. >85 forks analyzed per condition in each experiment; t-test. **(D)** Quantification of n=4 DNA content FACS experiments depicted in Fig 5B. G1, lovastatin G1-arrested cells. **(E)** Quantification of EdU-pulse labelled cells at 17 hrs by flow cytometry from n=3 experiments, Fig. 5C. Cells after 5-Ph-IAA addition (red); control cells (black) overlaid on FACS plot, with gating for S phase, EdU positive cells indicated. **(F)** Immunoblot of CDK2 levels in total cell extracts, accompanying Fig 5A. Loading control, Histone H2B. **(G)** 5 µM 5 Ph-IAA does not affect cell cycle progression in parental CMV-OsTIR1 (F74G) cells synchronized in parallel. Datapoints, n=3 experiments. Mean±SEM.

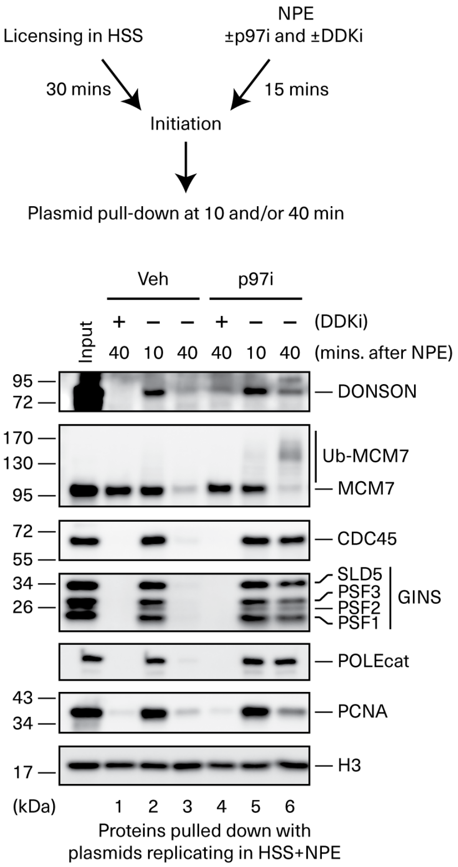

Fig. S17. DONSON associates with terminated CMGs. Top, depiction of experimental scheme. Bottom, plasmid DNA was incubated in the indicated egg extracts. At the specified times following NPE addition, chromatin was recovered and blotted for the indicated proteins. Replication termination and unloading of the CMG helicase, which normally occurs by 40 mins after NPE addition, was prevented by the inhibition of p97. The results show that a fraction of DONSON remains bound to chromatin 40 mins after NPE addition in the presence of p97i (compare lanes 3 and 6). Ub-MCM7, ubiquitylated MCM7; p97i, NMS-873.

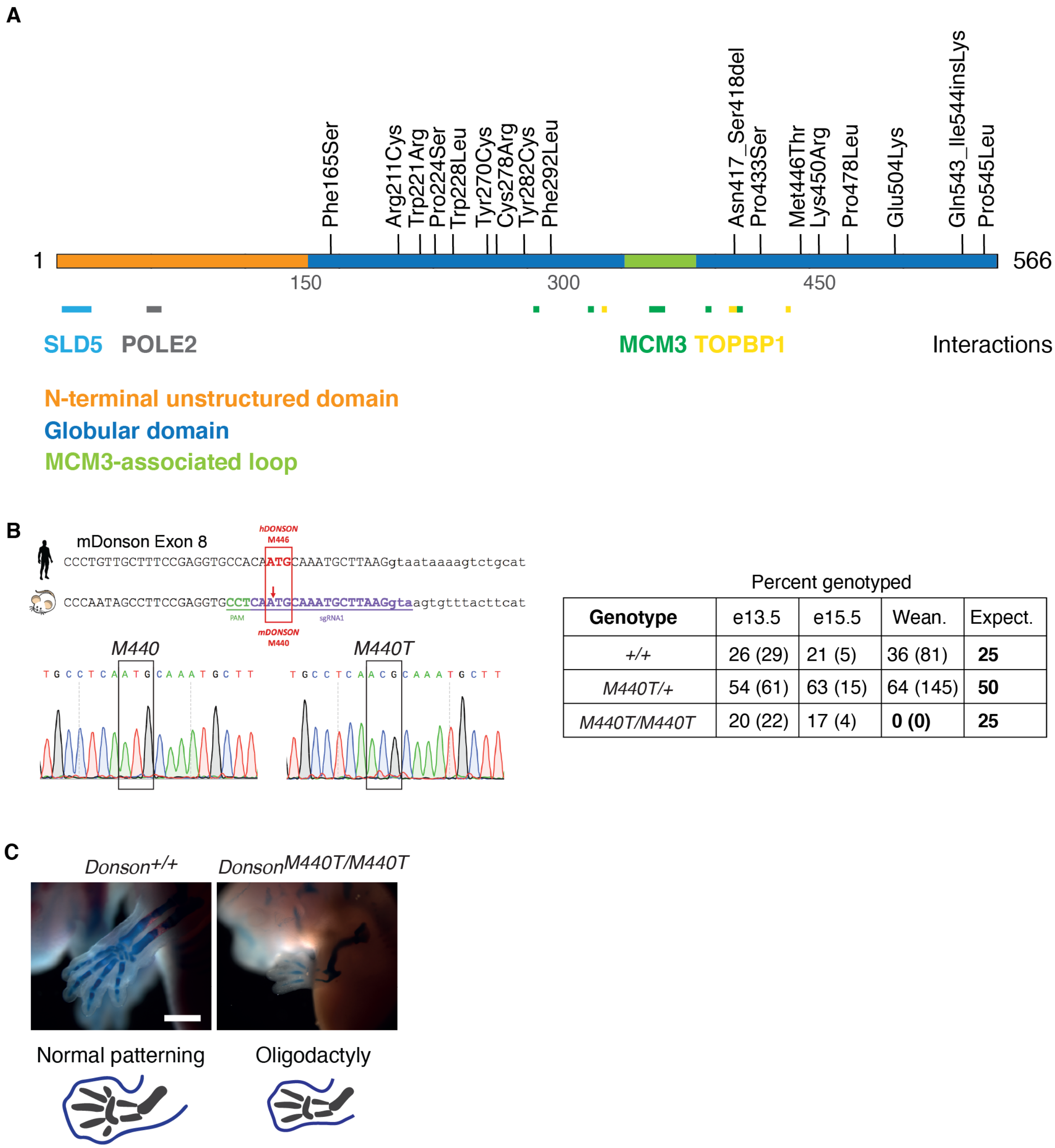

Genotype	e13.5	e15.5	Wean.	Expect.
+/+	26 (29)	21 (5)	36 (81)	**25**
M440T/+	54 (61)	63 (15)	64 (145)	**50**
M440T/M440T	20 (22)	17 (4)	**0 (0)**	**25**

Fig. S18. A mouse model of DONSON microcephalic dwarfism. (A) Location of human pathogenic missense mutations reported in DONSON, adapted from (*74*). N-terminal unstructured domain (orange), structured globular domain (blue) and MCM3-associated loop (green) are indicated. Below, human DONSON interaction surfaces predicted by AlphaFold with SLD5 (aa G7-R24); POLE2 (aa N78-R82), MCM3 (aa Q287, T311-G313, D357-E367, D394, K419) and TOPBP1 (aa E321, N413, N417, R442). Co-segregating variants as part of a 'Haplotype' in the discovery cohort reported by Reynolds, p.Ser28Arg, c.786-33A>G and p.Lys489Thr were omitted from the diagram. **(B)** Left, Schematic: CRISPR genome editing strategy to generate *M440T* substitution. *M440T* is the orthologous residue to human codon 446, that is homozygously mutated in DONSON cases with microcephalic dwarfism and limb abnormalities (c.1337T>C, p.M446T

(*23*)). Sequence electropherogram for DONSON exon 8, DNA extracted from embryos as indicated, confirming successful targeting. Right, genotyping of mice from $DONSON^{M440T/+}$ intercrosses. Homozygous mice are present at Mendelian ratios until late gestation but are not evident at weaning; number of mice in parentheses. This corresponds to the *in utero* and perinatal lethality phenotype associated with DONSON microcephaly-micromelia syndrome (*23, 24*). **(C)** $DONSON^{M440T/M440T}$ embryos have reduced digits and shortened long bones, consistent with micromelia; Alcian blue alizarin red staining. Scale bar 0.2mm.

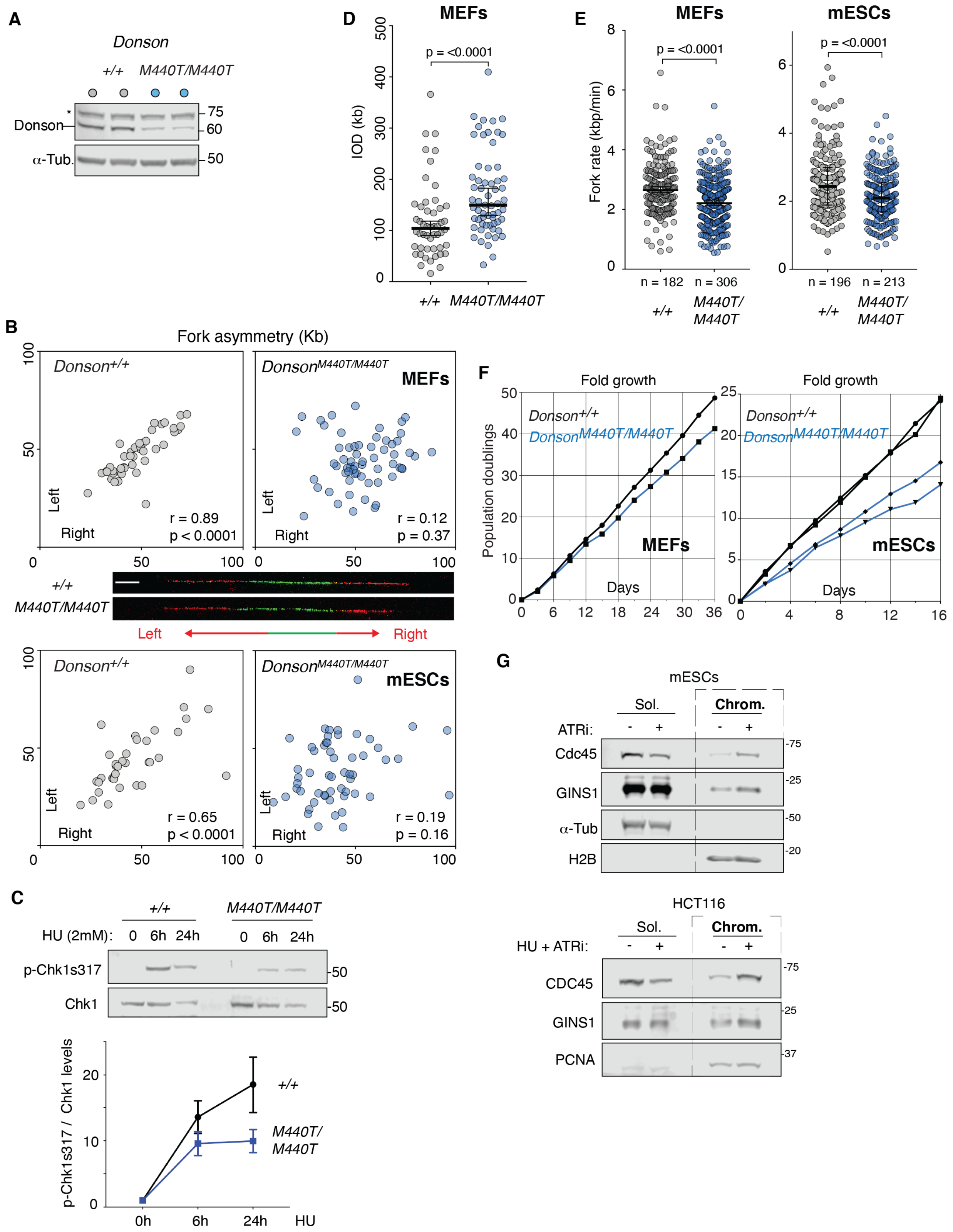

Fig. S19. Replisome number is reduced in embryonic *DONSON*$^{M440T/M440T}$ cells. (A) DONSON protein levels are reduced in *DONSON*$^{M440T/M440T}$ mESCs. Immunoblot, whole cell extracts. Loading control, alpha-tubulin (α-Tub). *, non-specific band. **(B)** DNA fibers from

DONSON$^{M440T/M440T}$ MEFs and mESCs have significant fork asymmetry; Left-Right ratio of ongoing bidirectional forks during second labelling pulse plotted in Kilobases (Kb); r, Pearson coefficient with p-value indicated. **(C)** ATR signaling is reduced in *DONSON*$^{M440T/M440T}$ mESCs. p-Chk1ser317 and total Chk1 immunoblots from total cell lysates of WT and *DONSON*$^{M440T/M440T}$ mESCs 6h and 24h after 2mM hydroxyurea (HU) treatment. Quantification, pChk1-ser317 normalized to total Chk1 protein plotted for 3 independent experiments; Mean±SEM, 2-way Anova. **(D)** Inter-origin distances (IOD) are significantly increased in *DONSON*$^{M440T/M440T}$ MEFs. **(E)** Fork speed is reduced in *DONSON*$^{M440T/M440T}$ MEFs and mESCs; number of fibers indicated. Median±95%confidence interval; U-test (D, E) **(F)** Cell proliferation is reduced in DONSON mESCs and MEFs. Doubling times in Wild-type and *DONSON*$^{M440T/M440T}$ in MEFs 18.15 and 21.4 hours, respectively; in mESCs, 16.4±1.0, and 25.7±3.6 hours, respectively. n=2 independent clones. *DONSON*$^{M440T/M440T}$ MEFs in B, D, E immortalized by TP53 knockout. (**G)** ATR inhibition increases GINS and Cdc45 levels on chromatin. Cell fractionation immunoblots of WT mESCs treated with vehicle or 2µM ATRi (AZD6738) for 3h. Representative of n=2 experiments. Below, GINS and Cdc45 levels are also increased by ATR inhibition after induction of replication stress. Immunoblot, WT HCT116 cells, released from G1 arrest into 1µM ATRi and 1mM hydroxyurea (HU).

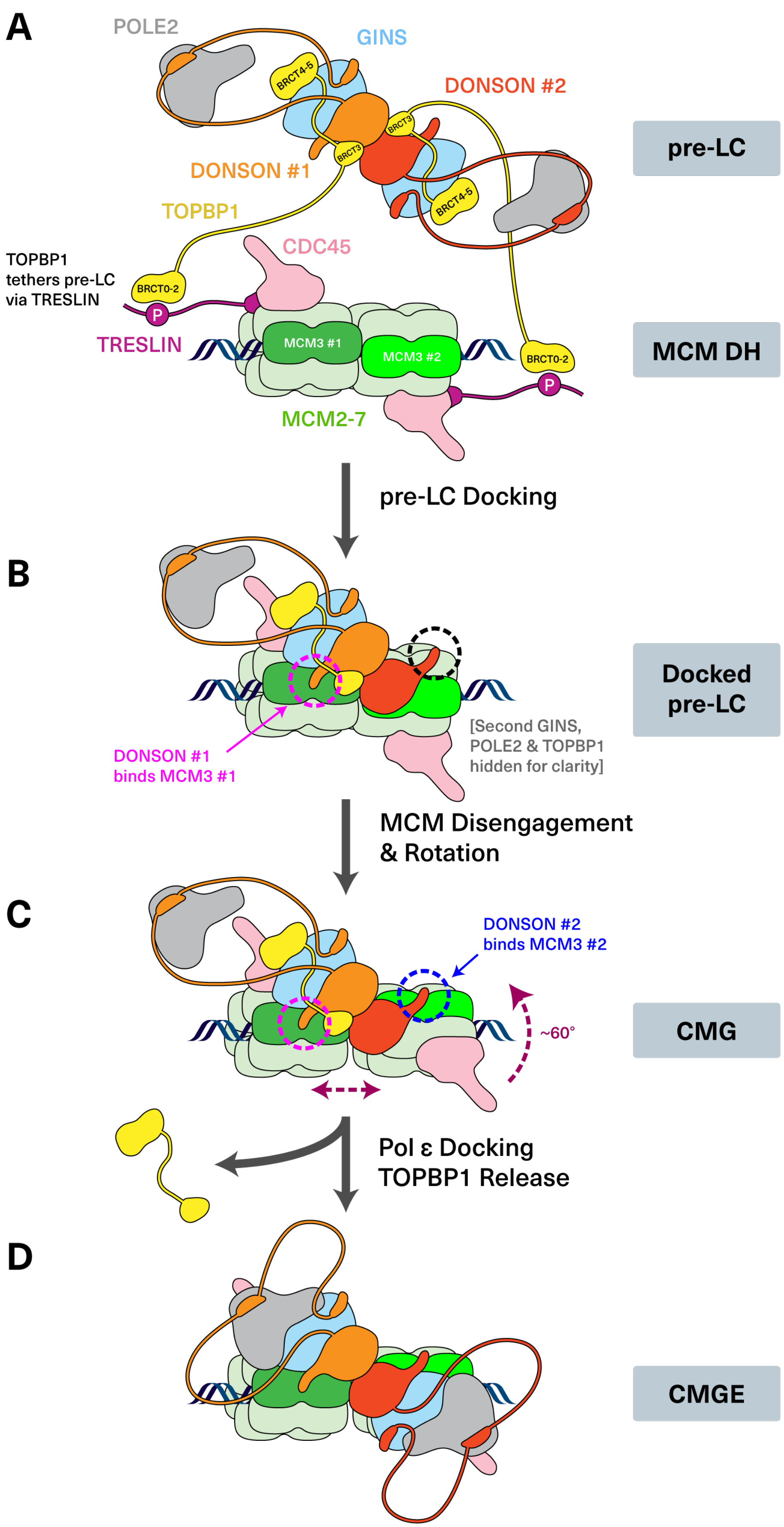
A
POLE2
GINS
DONSON #2
BRCT4-5
BRCT3
BRCT3
BRCT4-5
DONSON #1
TOPBP1
CDC45
TOPBP1
tethers pre-LC
via TRESLIN
BRCT0-2
P
TRESLIN
MCM3 #1
MCM3 #2
BRCT0-2
P
MCM2-7
pre-LC
MCM DH
pre-LC Docking
B
Docked
pre-LC
[Second GINS,
POLE2 & TOPBP1
hidden for clarity]
DONSON #1
binds MCM3 #1
MCM Disengagement
& Rotation
C
DONSON #2
binds MCM3 #2
~60°
CMG
Pol ε Docking
TOPBP1 Release
D
CMGE

Fig. S20. Model for vertebrate CMG assembly. Based on prior literature, AlphaFold structure predictions, and our biochemical and cell biological data, we propose the following model of CMG assembly, with an emphasis on DONSON's role. **(A)** Replication licensing establishes chromatin-bound MCM2-7 double hexamers ("MCM DH"), whose phosphorylation by DDK (not shown) allows recruitment of two TRESLIN-MTBP complexes (MTBP not shown) and two CDC45 molecules. An unstructured region in TRESLIN is phosphorylated by CDK (P), creating a binding site for the phosphopeptide-binding domain of TOPBP1 (BRCT0-2), which tethers the dimeric pre-LC to the MCM DH. **(B)** Once the pre-LC is tethered, one of the two DONSON molecules engages with one of the two MCM3 molecules within the MCM DH (dotted pink circle). Based on the geometry of the MCM DH (*75*) and the DONSON dimer, the second DONSON is in the wrong location to bind the second MCM3 (dotted black circle and Data S2). **(C)** We therefore speculate that binding of DONSON to the first MCM3 induces the partial disengagement of the two MCMs (double arrow). Clockwise rotation of the second MCM by ~60 degrees (single arrow) would allow DONSON engagement with the second MCM3 (dotted blue circle), leading to formation of two CMG complexes. Consistent with this mechanism, MCM double hexamer disengagement and a clockwise MCM register shift are observed during dual CMG assembly in yeast, but what triggers this rearrangement is unknown (*45*). Alternatively, binding of the first DONSON to the first MCM2-7 might destabilize the DONSON dimer, allowing engagement of the second DONSON with the second MCM2-7 without the need for MCM disengagement or rotation (not depicted). **(D)** The assembly of CMG induces cooperative binding of Pol ε to GINS and MCM2-7, generating two CMGE (CMG+Pol ε) complexes. Because POLE2 binds the same surface of GINS as TOPBP1's BRCT4-5 domains (fig. S9D; (*36*)), Pol ε docking destabilizes TOPBP1-GINS (fig. S10) (*36*), likely contributing to TOPBP1 dissociation.

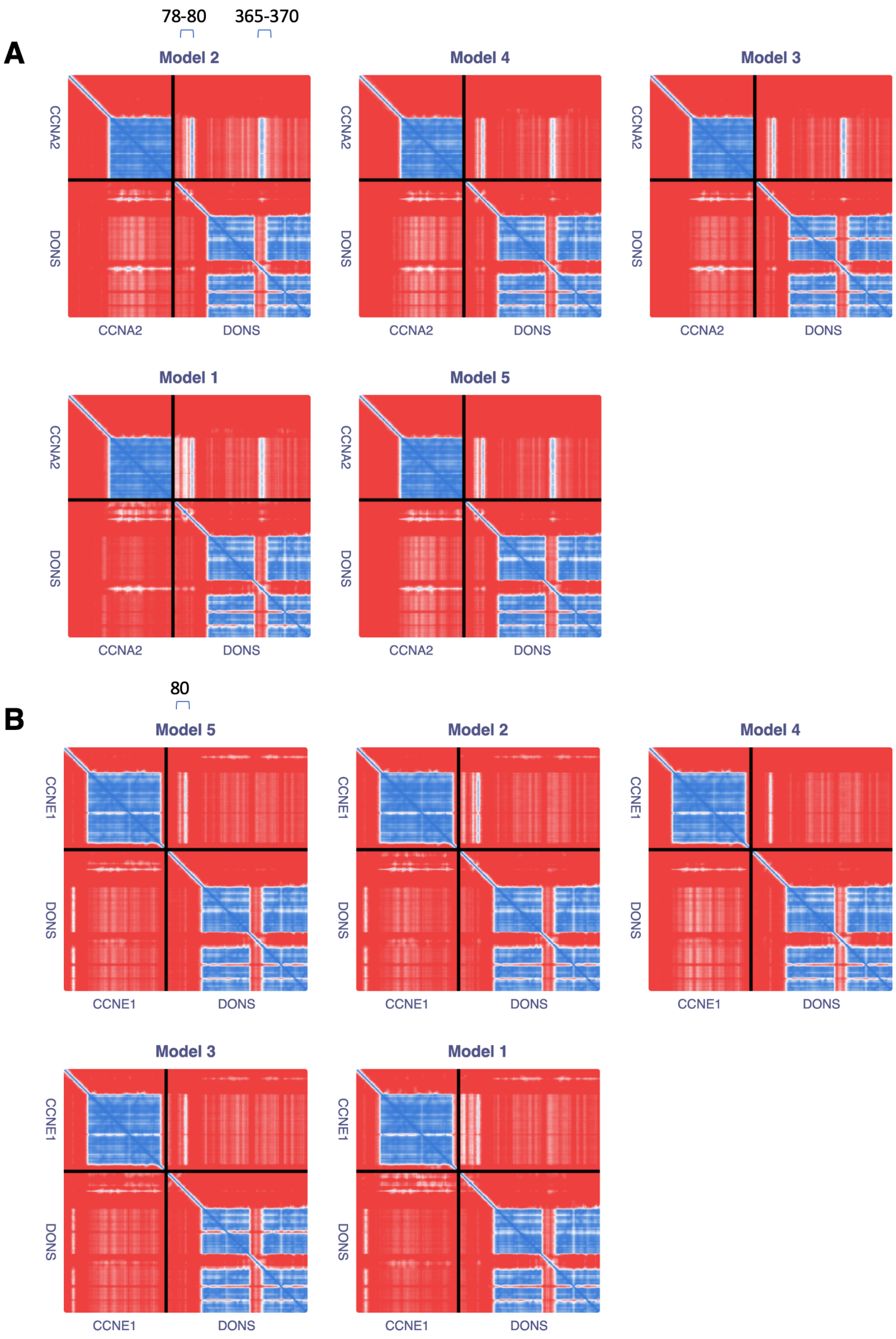

Fig. S21. Predicted alignment error (PAE) plots for DONSON and Cyclin A and Cyclin E (all human proteins). PAE plots generated by the five AF-M models for the complex of DONSON with **(A)** Cyclin A (CCNA2) and **(B)** Cyclin E (CCNE1). As indicated by the brackets, DONSON is predicted to interact with Cyclin A using residues 78-80 and with Cyclin E using residue 80, which corresponds to the region in *Xenopus* DONSON (residues 65-72) that we showed mediates binding to POLE2. However, the predicted binding interface with the cyclins is less extensive than for POLE2. Importantly, *Xenopus* DONSON co-IPs efficiently with POLE2 (fig.

3A), indicating that cyclins do not block DONSON's binding to Pol ε. DONSON is also predicted to bind Cyclin A using residues (365-370) adjacent to those predicted to interact with MCM3. However, when we used AF-M to fold human DONSON with Cyclin A and MCM3, DONSON interacted exclusively with MCM3. Additional work will be required to assess whether DONSON's predicted interactions with cyclins are physiologically relevant.

Data S1: ChimeraX session for the structure shown in fig. S4A. For details of structure generation, see "AlphaFold-based modeling of protein structures" section in Materials and Methods. For residue coloring, see the legend to fig. S4A. Because we folded only the 105 amino acid BRCT3 domain of TOPBP1 in the DONSON-DONSON-TOPBP1 structure (ID4), residues 1 and 105 of the BRCT3 domain correspond to residues 343 and 447 of full length TOPBP1, respectively. Note that the DONSON-POLE2 structure (ID2) was not aligned to the other structures and should be viewed on its own to visualize the interaction of POLE2 with DONSON amino acids 65-72, which are connected to the rest of DONSON via intrinsically disordered regions.

Data S2: ChimeraX session showing a DONSON dimer docking onto the MCM double hexamer (all proteins human). The two copies of DONSON (unstructured N-terminus hidden) are shown in orange and red, and the MCM3-binding helix (residues 355-369) in each is colored magenta. MCM3s are colored dark green whereas other MCMs are light green. The structure shows that when the MCM3-binding helix of one DONSON (orange) is engaged with one MCM3, the other DONSON (red) cannot engage the second MCM3. Engagement would require the MCM2-7 double hexamer to undergo major structural rearrangements including separation of the two MCMs and clockwise rotation of the right MCM2-7 relative to the left MCM2-7. To generate this structure, the predicted DONSON-MCM3 structure (ID2 in the ChimeraX session; residues 1-149 of DONSON and all of MCM3 hidden) was aligned on one MCM3 in the human MCM double hexamer cryo-EM structure (ID1; PDB: 7W1Y), and DONSON was colored orange. Next, the predicted DONSON dimer (ID3; residues 1-149 hidden) was aligned on the DONSON-MCM3 structure, revealing the position of the second DONSON relative to the second MCM3.
